# Single-Cell Approaches Define the Murine Leptomeninges: Cortical Brain Interface as a Distinct Cellular Neighborhood Composed of Neural and Non-neural Cell Types

**DOI:** 10.1523/ENEURO.0046-25.2025

**Published:** 2025-08-22

**Authors:** Sarah N. Ebert, Christine Eisner, Konstantina Karamboulas, Louis-Philippe Bernier, David R. Kaplan, Brian A. MacVicar, Freda D. Miller

**Affiliations:** ^1^Neuroscience Graduate Program, University of British Columbia, Vancouver, British Columbia V6T 1Z3, Canada; ^2^Michael Smith Laboratories, University of British Columbia, Vancouver, British Columbia V6T 1Z4, Canada; ^3^Djavad Mowafaghian Centre for Brain Health, University of British Columbia, Vancouver, British Columbia V6T 1Z3, Canada; ^4^Program in Neurosciences and Mental Health, Hospital for Sick Children, Toronto, Ontario M5G 0A4, Canada; ^5^Departments of Medical Genetics, University of British Columbia, Vancouver, British Columbia V6T 1Z3, Canada; ^6^Psychiatry, University of British Columbia, Vancouver, British Columbia V6T 1Z3, Canada; ^7^Department of Molecular Genetics, University of Toronto, Toronto, Ontario M5S 1A8, Canada

**Keywords:** astrocytes, layer 1 cortex, meninges, spatial transcriptomics

## Abstract

The interface barrier between the brain surface and the adjacent meninges is important for regulating exchanges of fluid, protein, and immune cells between the CNS and periphery. However, the cell types that form this important interface are not yet fully defined. To address this limitation, we used single-cell RNA sequencing (scRNA-seq) and single-cell spatial transcriptomics together with morphological lineage tracing and immunostaining to describe the cell types forming the interface barrier of the adult murine cortex. We show that the cortical interface is composed of three major cell types, leptomeningeal cells, border astrocytes, and tissue-resident macrophages. On the nonparenchymal side, the interface is composed of transcriptionally distinct PDGFRα-positive leptomeningeal cells that are intermingled with macrophages. This leptomeningeal layer is lined by a population of transcriptionally distinct border astrocytes. The interface neighborhood is rich in growth factor mRNAs, including many leptomeningeal ligands predicted to act on both the border astrocytes and macrophages. On the CNS side of the interface is the relatively cell-sparse cortical layer 1 containing interneurons, microglia, parenchymal astrocytes, oligodendrocyte precursor cells, and oligodendrocytes. Except for the border astrocytes, layer 1 cells are not closely associated with the interface, suggesting that secreted ligands may be the major way the brain interface communicates with the underlying cortical parenchyma. Thus, our data provide a molecular/cellular resource describing the brain interface cell types and their interactions, thereby enabling future studies investigating how this distinct cellular compartment regulates CNS:periphery interactions.

## Significance Statement

Recent years have seen significant progress in identifying the diverse cell types within the meningeal space. However, the mechanisms by which these cells interact with glial and neuronal cells in layer 1 of the adult murine cortex remain poorly understood. During development, communication between radial precursors and meningeal layers is crucial for proper brain formation, but the role of this interaction in adulthood is still unclear. Additionally, how resident immune cells in the leptomeningeal space signal to layer 1 cortical cells or meningeal mesenchymal cells during homeostasis remains an open question. Understanding the identity, location, and interactions of these cells is essential for unraveling the complex dynamics at this critical brain interface.

## Introduction

The interface between the brain surface and the periphery ensures maintenance of a distinct neural environment by acting as a selective barrier regulating the passage of fluid, proteins, and inflammatory cells. On the superficial side of this interface are the meninges, a complex tissue composed of blood vessels, nerves, immune cells, and several distinct populations of mesenchymal cells/fibroblasts (Rua and McGavern, 2018; Como et al., 2023; Betsholtz et al., 2024). The superficial-most meningeal layer is the dura, which is adjacent to the skull and functions as a connective tissue stroma (Rustenhoven et al., 2021; Kolabas et al., 2023). Closer to the brain are the leptomeninges, which are composed of arachnoid and arachnoid barrier cells, and, immediately adjacent to the brain, pial cells (DeSisto et al., 2020; Como et al., 2023; Betsholtz et al., 2024). In humans, these distinct layers have long been characterized anatomically, but we have only recently begun to understand the meninges at the molecular/cellular level, largely as a consequence of single-cell transcriptional studies (Wang et al., 2022; Kearns et al., 2023; Degenhard et al., 2024; Sun et al., 2025). Nonetheless, we still do not understand how leptomeningeal cells interact with CNS cells to form the brain:periphery interface, nor do we fully understand what happens when this interface is broached following traumatic injury, viral and bacterial attack, neurodegenerative disorders, or neurosurgery (Roth et al., 2014; Turtzo et al., 2020; Aydin et al., 2023; Derk et al., 2023; Ewing-Crystal et al., 2024).

What then do we know about the brain interface cells and how might we obtain a better understanding of their characteristics? We know that the adult brain interface includes leptomeningeal pial cells on the nonparenchymal side and astrocytes on the CNS side. One idea is that this astrocyte:pial cell interaction may be conceptually analogous to the much better-studied astrocyte:vasculature interaction. Indeed, several recent studies demonstrated that leptomeningeal cells are associated with large blood vessels that penetrate the brain from the outside (Bonney et al., 2022; Jones et al., 2023; Pietilä et al., 2023). However, there is little direct evidence that this astrocyte:pial cell interaction participates in blood–brain barrier function. Instead, several studies indicate that pial cells function as part of a brain surface signaling center, at least during embryogenesis when they provide a basal anchor for cortical radial precursors and secrete ligands that regulate multiple aspects of cortical development (Sievers et al., 1994; Hartmann et al., 1998; Choe et al., 2012; 2014; Izen et al., 2018; Dasgupta and Jeong, 2019; Zelco et al., 2021; Como et al., 2023). Whether adult pial cells play similar roles is unclear, although some studies suggest that they might. For example, when cortical leptomeningeal cells were removed locally, this caused perturbations in both cortical layer 1 and cortical midline structures suggesting that leptomeningeal cells may play multiple roles in the adult brain (Nikouei et al., 2024).

These studies highlight the importance of acquiring a molecular/cellular understanding of adult brain interface cells and their interactions. Here, we have addressed this by creating a single-cell RNA sequencing and spatial transcriptomic resource focused on the cortical brain interface and adjacent cortical layer 1. Our data define three distinct cell types within the brain interface: leptomeningeal cells, border astrocytes that are transcriptionally distinct from parenchymal astrocytes, and a population of tissue-resident border macrophages. We show that these three cell types comprise a tightly organized cellular neighborhood and that this neighborhood is not closely associated with any layer 1 cortical cell type other than the border astrocytes. Moreover, we identify the ligands and ligand receptors expressed by the three interface cell types and use these to predict paracrine interactions within the interface neighborhood. Thus, our data provide a molecular/cellular resource describing the brain interface cell types and their potential interactions, thereby enabling future studies asking about the functions of this distinct cellular compartment during homeostasis and following injury.

## Materials and Methods

### Animals

All animal protocols were approved by the Animal Care Committees of either the Hospital for Sick Children or the University of British Columbia in accordance with national animal care policies. Animals had *ad libitum* access to rodent chow and water and were housed in a temperature- and humidity-controlled environment on a 12 h light/dark cycle. All mice were healthy with no obvious behavioral phenotypes. For all studies, adult (8–16-week-old) mice of either sex were used, and mice were randomly allocated to experimental groups. Wild-type C57BL6 mice were purchased from Charles River Laboratories (strain codes: 027) or Jackson Laboratories (strain code: 000664). *PdgfraCreERT2* (B6.129S-*Pdgfra^tm1.1(cre/ERT2)Blh/^*J; JAX stock #032770) RRID:IMSR_JAX:032770 (Chung et al., 2018), *R26-LSL-TdTomato* (B6.Cg-*Gt(ROSA)26Sor^tm9(CAG-tdTomato)Hze^/*J; JAX stock #007909) RRID:IMSR_JAX:007909 (Madisen et al., 2010), and *Tmem119CreERT2* (C57BL/6-*Tmem119^em1(cre/ERT2)Gfng^*/J; JAX stock #031820) RRID:IMSR_JAX:031820 (Kaiser and Feng, 2019) were obtained from Jackson Laboratories. All mice were bred and genotyped as recommended by Jackson Laboratories.

For experiments involving inducible Cre recombinase mouse strains, tamoxifen (Sigma) was dissolved in a sunflower oil at 30 mg/ml. Mice were injected intraperitoneally with 3 mg/day for 5 consecutive days, followed by a 10 d washout period and then collected for imaging.

### Tissue preparation, immunostaining, and microscopy

For immunostaining analyses mice were perfused transcardially with ice-cold 1× PBS and then with 4% PFA. The head, lower jaw, and skin were removed from the skull, and the snout removed just rostral to the eyes. Excess muscle tissue was also removed from the cranium. Finally, the ventral portion of the upper palate was removed exposing the ventral surface of the brain. The skull and brain were postfixed in 4% PFA for 18–24 h at 4°C and then washed in PBS. To decalcify the skull, the tissue was incubated in 0.5 M EDTA, pH 8.0, at 4°C for 4 d or until bone was sufficiently soft. Tissue was then incubated in 15% sucrose for 48 h at 4°C, followed by an incubation in 30% sucrose for 48 h or until tissue had sunk. Tissue was then incubated in OCT for 4 h at 4°C with slow rotation and snap fixed in fresh OCT. In the case of the CX26 antibody, it was incompatible with decalcification, so the dura was dissected directly on top of the brain post perfusion and fixation, incubated in sucrose and OCT as above, and then snap fixed in fresh OCT. Brains were sectioned sagittally, and tissue was collected from the M1 region line preserving the orientation of skull, meninges, and brain. Sections were 12–14 μm thick and stored at −80°C until immunostained.

For immunostaining, sections were dried for 15 min at 37°C or until all condensation disappeared, incubated in 1× PBS for 10 min to rehydrate, and then blocked using 5% BSA (Jackson ImmunoResearch) containing 0.3% Triton X-100 (Fisher) in PBS for 1 h at room temperature. Sections were incubated with primary antibodies diluted in 5% BSA in PBS overnight at 4°C. Sections were washed three times with PBS prior to application of appropriate secondary antibodies at optimized concentrations (see below, Antibodies). Following a 1 h room temperature incubation in the dark, sections were washed three times with PBS, counterstained with DAPI (1:1,000) for 5 min at room temperature, and washed a further three times with PBS. Coverslips were applied to tissue with Vectashield (VectorLabs) and sealed.

Images were collected with either a Zeiss Axio Imager M2 system with an X-Cite 120LED light source, Apotome 3 and C11440 Hamamatsu camera, or a Zeiss LSM900 with an Airyscan 2 detector. Images were processed using Fiji (Schindelin et al., 2012; RRID: SCR_002285). Gamma adjustments for GFAP immunostaining in Figure 3 were used to brighten processes against very bright cell bodies (0.75). Similarly, for *Tmem119CreERT2*:Tdtomato strain imaging in the same figure, the Tdt channel was also adjusted (0.75).

### Single-cell isolation and scRNA-seq

For single-cell isolation of meningeal cells, tissue was isolated from the inside of the skull (bone-associated or BA) and the cortical surface of the brain (cortical surface-associated or CSA). For the former, the top of the skull above the cortex with associated tissue was removed from the brain surface, and the meningeal tissue was carefully removed from the inner side of the skull under a microscope in ice-cold PBS. The tissue was chopped using a scalpel and collected into a 15 ml Falcon tube containing 2 ml of collagenase D (1.5 U/ml; Roche ref 11088882001)/dispase II (2.4 U/ml; Roche ref 4942078001) + 10 µl/ml CaCl2 (stock 250 mM) and incubated at 37°C with rotation/agitation for 45 min or until digested. Every 15 min the sample was checked and vortexed. Post digest, the cells were triturated with a P200 Pipetman followed by a polished glass pipette, topped up to 15 ml volume with HBSS + 2 mM EDTA. It was then filtered through a 70 μM filter and spun down for 7 min at 800 × *g*. The supernatant was removed and the pellet resuspended in 1 ml of 0.2% BSA in HBSS for FACS sorting and sequencing.

For the cortical surface-associated digest, the top half of the dorsal cortex was glued onto a 1-cm-diameter circular coverslip in a 24-well plate and rinsed with HBSS, and the HBSS was then replaced with 1–1.5 ml of collagenase dispase solution as above. The plate was incubated at 37°C with rotation/agitation for 30–45 min or until digested. Every 15 min, the sample was checked, and the well solution was “washed” over the brain five times with a pipette for each sample. Once digestion was complete, the supernatant from each well was combined into a 15 ml Falcon tube, triturated with a P200 Pipetman and polished glass pipette, and topped up to 15 ml with HBSS + 2 mM EDTA. The sample was filtered through a 70 μM filter, spun down for 7 min at 800 × *g*, supernatant removed, and resuspended in 1 ml of 0.2% BSA in HBSS for FACS sorting and sequencing.

For all samples, viable cells were incubated in DRAQ5 (1:3,000) and propidium iodide (PI; 1:1,000) and sorted by flow cytometry. Cells that were DRAQ5-positive and PI-negative were collected in DMEM, ʟ-glutamine, and B27 and submitted for single-cell isolation and processing using the 10x Genomics Chromium system as per the manufacturer’s guidelines. The resultant libraries were sequenced on an Illumina NextSeq500. FASTQ sequencing reads were processed, aligned to wild-type mouse genome (mm10), and converted to digital gene expression matrices using the Cell Ranger count function within the Cell Ranger Single-Cell Software Suite (RRID: SCR_017344) with settings as recommended by the manufacturer (https://support.10xgenomics.com/single-cell-gene-expression/software/overview/welcome; Zheng et al., 2017). Two bone-associated and four cortical surface-associated samples were sequenced that each included tissue from equal numbers of mice of each sex. The two bone-associated runs included tissue from eight mice each. The four cortical surface-associated runs included tissue from 6, 8, 10, and 8 mice total.

### scRNA-seq analysis

Dataset count matrices were processed using a previously published pipeline (Innes and Bader, 2019; Borrett et al., 2020; Willis et al., 2025). In brief, datasets were filtered to remove low expressing cells and cells with a high mitochondrial gene expression profile. Using R Project for Statistical Computing (version 4.1,2; R Core Team, 2021), datasets were normalized using scran (version 1.28.0), and data was transferred into Seurat (version 4.0.1, RRID:SCR_016341; Lun et al., 2016; Hao et al., 2021). PCA was performed using highly variable genes and UMAPs generated using the top principal components detected in the datasets. Once clustered, all datasets were subsetted to remove clusters with low transcript counts, and the raw count matrices for the selected cells were run back through the pipeline omitting filtering steps. Cell cycle scores were computed as part of the pipeline using Cyclone, part of the scran package. Two-dimensional t-SNE projections were generated using the top principal components (RunTSNE, Seurat). The same principal components were also used to execute SNN-Cliq-inspired clustering (FindClusters, Seurat) with iteratively increasing resolution until the number of differentially expressed genes [FindMarkers, Seurat, where *p* < 0.01 familywise error rate (FWER), Holm method] between the most similar clusters reached ∼30 genes. All individual or merged datasets were analyzed with the most conservative resolution selected based on clustering that best aligned with the expression of well-defined markers for each cell type.

We used gene expression overlays on Uniform Manifold Approximation and Projection (UMAP) and annotated clusters and cell type identities based on the following markers: *Pdgfra*, *Sox10*, *Cspg4*, and *Enpp6* for oligodendrocyte precursor cells (OPCs; Dennis, Wang et al., 2024; Willis et al., 2025); *Mbp*, *Mog*, *Mag*, and *Sox10* for oligodendrocytes (Dennis, Wang et al., 2024; Willis et al., 2025); *Pdgfrb*, *Myh11*, *Mylk*, and *Acta2* for vascular smooth muscle cells (VSMCs); *Pdgfrb*, *Rgs5*, *Cspg4*, and *Kcnj8* for pericytes (Vanlandewijck et al., 2018); *Ptprc*, *Cd68*, and *Cd44* for all immune cells (Mahmud et al., 2022); *Lyve1*, *Mrc1*, *Dab2*, and *Clec10a* for border macrophages (Sankowski et al., 2024); *Clec4n*, *Folr1*, *Ccr2*, and *Lgals3* for dural macrophages (Sankowski et al., 2024); *Cxcr2* for neutrophils (Marin-Esteban et al., 2021); *Cd19* for B cells (Wang et al., 2012); *Nkg7*, *Cd3e*, *Cd4*, and *Cd8a* for T cells (Wen et al., 2022; Mullan et al., 2023); *Aif1*, *Sall1*, *Tmem119*, *Cx3cr1*, *Aif1*, and *Iba1*for microglia (Sankowski et al., 2024); *Aqp4*, *Slc1a3*, *Arxes2*, and *Cxcl14* for astrocytes (Willis et al., 2025); *Myoc* and *Gfap* for border astrocytes (Hasel et al., 2025); *Plp1*, *Sox2*, *Ngfr*, *Sox10*, and *Sema3d* for Schwann cells (Toma et al., 2020); *Pecam1*, *Plvap*, *Ly6c1*, and *Cdh5* for endothelial cells (Vanlandewijck et al., 2018); *Ttr* and *Foxj1* for choroid plexus (Pietilä et al., 2023; Willis et al., 2025); *Pdgfra*, *Col1a1*, *Gjb6*, *Gjb2*, *Bmp7*, *Bmp6*, *Wnt5a*, *Lama1*, and *Ptgds* for leptomeningeal cells (De Sisto et al., 2020; Pietilä et al., 2023); *Pdgfra*, *Col1a1*, *H19*, *Matn4*, *Clec11a*, and *Shisa3* for dural cells (De Sisto et al., 2020; Pietilä et al., 2023); *Gad1* and *Gad2* for interneurons; and *Slc17a7* and *Slc17a6* for excitatory neurons (Tremblay et al., 2016).

### Gene signatures

**Figure 1.**
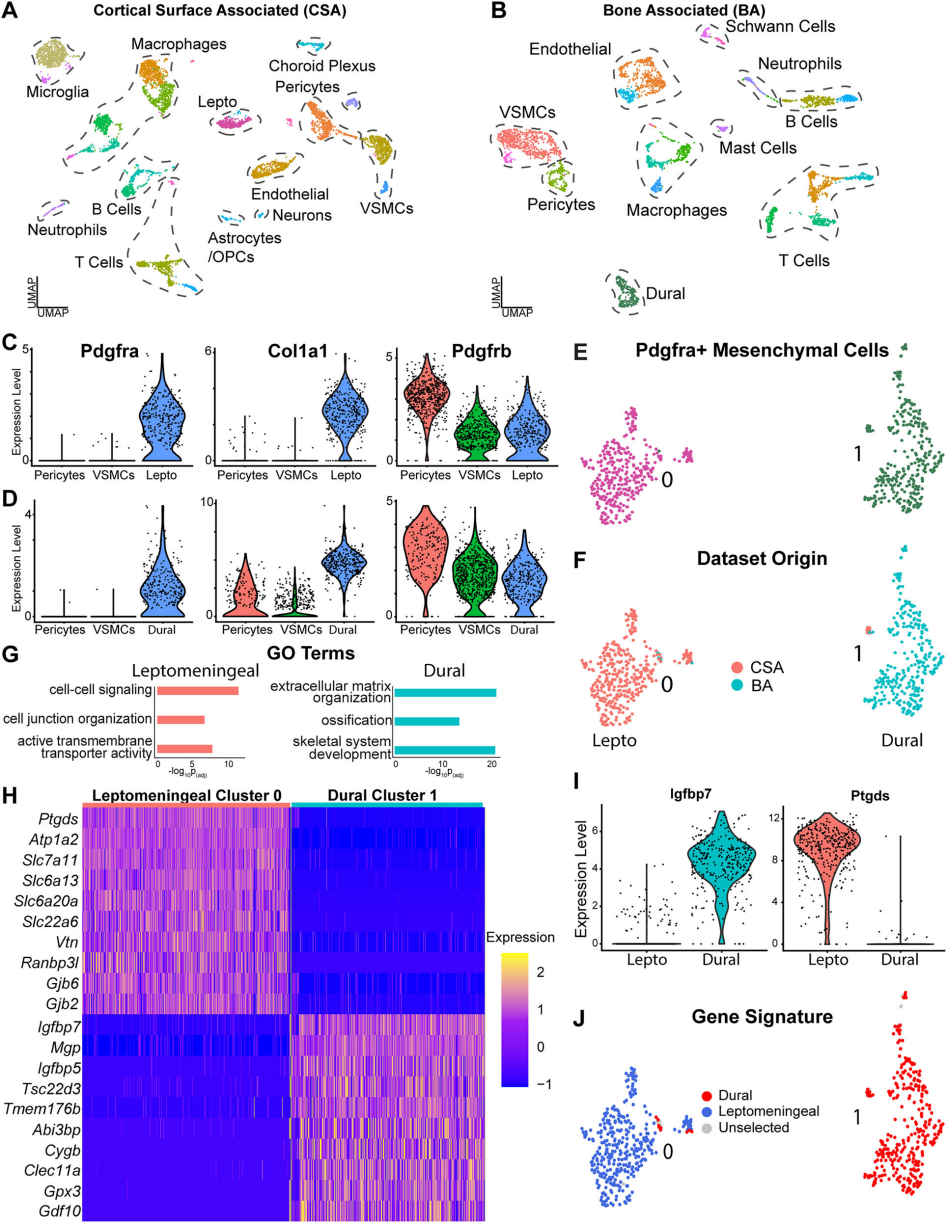
scRNA-seq identifies distinct populations of cortical brain interface versus bone-associated cells. Also see Extended Data Figures 1-1, 1-2, and 1-3. ***A***, ***B***, UMAP visualization of all transcriptomes identified in the cortical surface-associated (CSA; ***A***) or cortical bone-associated (BA; ***B***) datasets as analyzed by scRNA-seq. Shown are the mergers of four independent surface-associated runs (***A***) and two independent bone-associated runs (***B***). The merged CSA dataset in (***A***) was batch corrected using one iteration of Harmony. Transcriptionally distinct clusters are color coded, and plots are annotated for cell types corresponding to these clusters, as determined by analysis of well-characterized marker genes. VSMCs, vascular smooth muscle cells; Lepto, leptomeningeal cells; Dural, dural cells. ***C***, ***D***, Violin plots showing expression of *Pdgfra*, *Pdgfrb*, and *Col1a1* in leptomeningeal (***C***; colored blue) and dural (***D***; colored blue) cells relative to the *Pdgfra*-negative pericytes (colored red) and VSMCs (colored green). Dots represent expression levels in individual cells. ***E***, ***F***, *Pdgfra*-positive cell transcriptomes were subsetted from the surface-associated and bone-associated datasets shown in ***A*** and ***B*** and merged together. Panel ***E*** shows the cluster UMAP, where transcriptionally distinct clusters are colored and numbered and ***F*** shows the dataset of origin for each transcriptome. CSA, cortical surface associated; BA, bone associated. ***G***, Gene ontology was performed on the differentially expressed genes identified in the comparison between leptomeningeal and dural cells as defined in ***F*** (>1.3-fold average change, *p*adj < 0.05; Extended Data Figs. 1-2 and 1-3). Shown are selected categories (*y*-axis) and the adjusted *p* value (*x*-axis). The peach-colored bars are categories enriched in leptomeningeal cells and the turquoise enriched in dural cells. ***H***, Single-cell heatmap of select mRNAs that were differentially expressed (Extended Data Fig. 1-2) in the comparison between the leptomeningeal cells in cluster 0 and the dural cells in cluster 1 from the UMAPs in ***E*** and ***F***. Every row represents expression in an individual cell, and levels are color coded as per the adjacent key. ***I***, Violin plots showing relative expression levels of two mRNAs, *Ptgds* and *Igfbp7*, that were identified in the differential gene expression analysis as highly enriched in leptomeningeal (colored red) versus dural (colored turquoise) cells, respectively. Dots represent expression levels in individual cells. ***J***, Transcriptional signatures for the putative leptomeningeal and dural cells were defined using the 50 most differentially expressed mRNAs in the leptomeningeal versus dural cell comparison (Extended Data Fig. 1-2). Shown is a UMAP as in ***E*** highlighting cells expressing a transcriptional signature score of >0.5 for one or the other cell type.

To develop gene signatures for dural versus leptomeningeal cells, *FindMarkers* was run with parameters consistent with developing the DEG tables [DEG lists were defined as those expressed in >10% of cells in either group with a Bonferroni adjusted *p* value < 0.05 (adj. *p* value) and ≥1.3 average log2 fold change (Avg log2FC)]. From this list, the top 50 genes by adjusted *p* value were selected and used as a signature (AddModuleScore). Cells were highlighted as shown in Figure 1*J* if the module score was >50% in each condition. Similar parameters were used for the border astrocyte gene signature with the top 20 genes being selected from the DEG list.

### Pearson’s correlation analysis

Pearson’s correlation of scRNA-seq data was performed between leptomeningeal and dural cells by averaging the expression of each gene across all cells and then determining the Pearson’s correlation coefficient with the cor.test function in R.

### Dataset merger and batch correction

To generate the merged cortical surface-associated datasets and the bone-associated datasets, all cells passing library thresholds were extracted from each dataset, and the raw transcriptomes were run through the pipeline as indicated above. Following PCA, batch correction was used for the cortical surface-associated datasets using one iteration of Harmony to correct for sequencing differences (Korsunsky et al., 2019). No batch correction was needed for bone-associated datasets. When subsetting *Pdgfra*-positive clusters to generate the merged meningeal mesenchymal cell dataset, leptomeningeal and dural meningeal cells were extracted and merged and raw transcriptomes run through the pipeline as indicated above. One iteration of Harmony was performed to correct for the aforementioned sequencing differences within the cortical surface-associated datasets.

For astrocyte datasets, a similar process was used for each of six previously published datasets (GEO GSE148611; Hasel et al., 2021). These were merged with astrocytes from the previously published P60 Grey Matter and P60 Cortex datasets (GEO GSE255405; Dennis et al., 2024) along with astrocytes from cortical surface datasets described in this manuscript. One iteration of Harmony was performed to correct for sequencing depth differences between datasets. Batch ID was assigned with all six Hasel et al. (2021) datasets under one batch and each subsequent dataset with its own Batch ID.

To analyze and merge the previously published adult mouse scRNA-seq dataset from Pietilä et al. (2023) (GEO GSE227713), we chose two brain surface scRNA-seq runs that best matched our digest parameters. In brief, these tissues were collected directly from the brain surface and underwent enzymatic digest prior to droplet-based scRNA-seq using the NextSeq 2000 platform. Further details are referenced in the methods section of Pietilä et al. (2023). We merged and processed these two datasets through our pipeline, with no need for batch correction. The cells termed BFB 1-5 in Pietilä et al. (2023) were then subsetted, reclustered, analyzed for their reported markers and annotated as in Pietilä et al. (2023), their Figure 1D (far left panel). Our reanalysis reproduced their results, with the clusters termed leptomeninges in Extended Data Figure 6-1*A* corresponding to BFB 1-3. We then merged all transcriptomes from this subsetted, reprocessed dataset with the leptomeningeal and dural cell transcriptomes isolated and characterized here (Extended Data Fig. 6-1*B,C*). We performed two rounds of Harmony batch correction to account for differences in sequencing depth (as seen in Extended Data Fig. 6-1*D*). Batch ID was assigned with both Pietilä datasets as one group and the CSA and BA datasets as one group to remain consistent with other analyses in this paper.

For the merged leptomeningeal cell, dural cell, and border astrocyte dataset, and for the merged leptomeningeal cell, border astrocyte, and border macrophage dataset, appropriate clusters were extracted from the original datasets of origin, merged together as described above, and one iteration of Harmony was performed to correct for sequencing differences.

### Ligand–receptor analysis

Ligand–receptor analysis was performed essentially as described in Willis et al. (2025) using a curated ligand–receptor database and the CCINX package (Toma et al., 2020). Ligands and receptors were included when they were detectably expressed in >5% of the relevant cell types. Cytoscape (v3.9.1, SCR_003032) was used to visualize the predictive ligand–receptor communication models, where ligands included in the model are presented in a central panel of nodes and edges connecting them to their source and target cell type (Shannon et al., 2003).

### Xenium in situ based single-cell spatial transcriptomics

Single-cell multiplexed in situ gene expression analysis was performed using the Xenium platform as described (Willis et al., 2025). Analysis was performed on adult C57Bl/6 mouse brain tissue with or without an accompanying dural dissection. We included sections from five adult C57Bl/6 mice for analyses with a probeset targeting 347 genes composed of the 10x Genomics Adult Brain Panel (247 genes targeted) plus a custom add-on probeset (WHMTAZ; 100 additional genes targeted as shown in Extended Data Fig. 4-3). Two of these mice were analyzed in the current study and three were previously published in Willis et al. (2025) (GEO GSE266689, sections GSM8647390, GSM8647391, GSM8647392). For the latter, the ROI examined here had not been previously analyzed. We also analyzed sections from four adult C57Bl/6 mice with a 480 gene custom probeset designed to distinguish different non-neural cell types including mesenchymal cells (10x Genomics, 9XXV3X; Extended Data Fig. 4-3). For both probesets, one to two sections per mouse were analyzed by Xenium. Thus, we analyzed 4–5 independent biological replicates, consistent with current standards in the field (Ma et al., 2024; Willis et al., 2025).

Following RNAse-free removal of brains, fresh tissue was flash frozen in OCT embedding matrix and stored at −70°C. Subsequently, 10 µm rostral coronal cryosections (for level, see Extended Data Fig. 4-1*A*) were mounted onto Xenium slides (chemistry v2) following 10x Genomics guidelines. All tissues were equilibrated to −21°C in a cryostat prior to sectioning. Each coronal section was collected within the M1 cortical region, rostral to the hippocampus. For slides prepared for the mesenchymal probeset, the dura was carefully dissected off the skull and remained with the brain tissue for embedding. Slides were stored at −70°C prior to subsequent preparation steps.

Sectioned tissue was processed according to the Xenium workflow protocol for fresh-frozen tissue. To fix and permeabilize the tissue, sections were incubated for 1 min at 37°C and then fixed in 4% PFA (Electron Microscopy Sciences) for 30 min at room temperature. Sections were washed in RNAse-free 1× PBS for 1 min (Thermo Fisher Scientific) and subjected to a series of permeabilization steps and PBS washes. Permeabilization steps included 1% SDS incubation for 2 min and a chilled 70% methanol incubation for 1 h. Following permeabilization, slides were washed twice with PBS and then placed in PBS/0.05% Tween-20 (PBS-T, Thermo Fisher Scientific). Sections were hybridized with either a brain probeset (BP) targeting 347 genes (Extended Data Fig. 4-3) or a mesenchymal probeset (MP) targeting 480 genes (Extended Data Fig. 4-3) in TE buffer, for 22 h at 50°C. Afterward, sections underwent several PBS washes and were incubated at 37°C in posthybridization wash buffer. Following a series of PBS-T washes, sections were incubated in Xenium ligation enzymes for 2 h at 37°C. Following multiple rounds of PBS-T washes, probe amplification was achieved by incubating sections for 2 h at 30°C in Xenium amplification enzyme solution. Sections were washed twice in TE buffer and stored overnight at 4°C. Autofluorescence quenching and nuclei staining were next performed. Sections were washed in PBS, incubated in reducing agent for 10 min, washed in 70 and 100% ethanol, and incubated in Xenium autofluorescence solution for 10 min. Sections were then washed three times in 100% ethanol, dried at 37°C for 5 min, and rehydrated via a series of PBS and PBS-T washes. Nuclear staining buffer was added to sections for 1 min, and sections were washed four times in PBS-T. Samples were loaded into the Xenium analyzer instrument (v1.7.6.0) and subjected to several cycles of reagent application, probe hybridization, imaging, and probe removal. Preprocessing of captured *Z*-stack images was performed using the Xenium on-board analysis pipeline. In brief, a custom Xenium codebook (described below) was used to decode imaged fluorescent puncta into transcripts, with each decoded fluorescence signature assigned a codeword that was associated to a target gene. Quality scores (Q-scores) were assigned to transcripts based on maximum likelihood codewords compared with the likelihood of other suboptimal codewords, to provide the confidence in each decoded transcripts assigned identity. Only transcripts with a Q-score ≥20 were included in downstream analysis. Negative control codewords (codewords that do not correspond to any probe) and negative control probes (probes included in the panel that do not match any biological sequence) were utilized to assess decoding accuracy and assay specificity, respectively. On-instrument cell segmentation was performed based on 3D DAPI morphology. Nuclei identification and cell boundaries were subsequently flattened into a 2D mask, cell IDs allocated to each identified cell, and transcripts assigned to cell IDs based on their *x*–*y* coordinates. The boundary for expansion around the nucleus was set for 2 μm manually for each section to account for the tight packing of cells at the brain interface.

### Single-cell spatial transcriptomic data analysis

Standardized Xenium output files were exported for downstream analyses. In addition, we downloaded and analyzed three datasets from Willis et al. (2025) (GEO GSE266689, sections GSM8647390, GSM8647391, GSM8647392) that were run using the same 347 gene brain probeset. These slightly more caudal sections were from control perfused brains (Extended Data Fig. 4-1*B*). Nonetheless, transcriptomes from the selected ROI merged well with the datasets generated from the more rostral fresh-frozen sections (Extended Data Fig. 4-1*C*). Data were visualized in Xenium Explorer (version 3.0, RRID: SCR_025847), with regions of interest (ROIs) defined using the freehand select tool and cell IDs exported (Janesick et al., 2023). ROIs were drawn to include the brain surface, and the cortex down to the bottom of layer 2. Transcript count, feature, coordinate, and cell ID data were imported to R and Seurat objects created (v5.0.1, RRID:SCR_016341) via the LoadXenium function (Hao et al., 2024). Subsets were then produced from object data using the previously exported cell IDs to include only cells contained within our ROI, and these regions were concatenated where multiple ROIs were specified on one section. All downstream Xenium analysis was conducted in R package Seurat. Initial quality checks assessed cells based on the number of detected genes and total transcript counts per cell. Low-quality cells with greater than +/−2.5 standard deviations from the mean in either of these parameters were excluded.

Filtered data were normalized using SCTransform and PCA based on highly variable genes to capture main sources of variation within the data and reduce dimensionality. To construct a nearest neighbor (SNN) graph, Seurat Find Neighbors was used with principal components identified through PCA. A Louvain clustering algorithm based in Seurat (Find Clusters) was applied to partition cells into distinct clusters based on transcriptomic profile. Further analysis was performed on the smallest, yet still biologically meaningful clustering resolution.

UMAPs and image dimensional reduction plots visualizing cell coordinates (ImageDimPlot) were employed to visualize the data. Cell clusters were annotated using well-defined marker genes for specific cell types. For slides analyzed with the brain probeset, leptomeningeal cells were identified with *Lama1*, *Slc22a6*, *Gjb2*, and *Dpp4* (DeSisto et al., 2020; Pietilä et al., 2023); astrocytes with *Aqp4* and *Gja1*; and border astrocytes specifically with *Gfap* and *Myoc* (Sofroniew, 2020*;* Hasel et al., 2025). Microglia were identified with *Tmem119*, *Cx3cr1*, and *Cd68* and border macrophages with *Cybb* and *Cd53* (Joost et al., 2019; Sankowski et al., 2024)*.* Excitatory neurons were identified with *Dpyd*, *Rspo2*, *Adamts2*, and *Cdh13* and various populations of inhibitory neurons with *Gad1*, *Gad2*, *Lamp5*, *Pvalb*, *Vip*, and *Sst* (Yao et al., 2021)*.* OPCs and oligodendrocytes were identified with *Olig1*, *Olig2*, and *Sox10*, and OPCs specifically with *Cspg4* and *Pdgfra* (Willis et al., 2025)*.* VSMCs were identified with *Acta2* and *Pdgfrb*, pericytes with *Cspg4* and *Pdgfrb*, and endothelial cells with *Pecam1* and *Cdh5* (Willis et al., 2025).

For the mesenchymal probeset, leptomeningeal cells were identified with *Pdgfra*, *Lama1*, *Slc22a6*, *Gjb2*, and *Dpp4* (DeSisto et al., 2020; Pietilä et al., 2023) and dural cells with *Pdgfra*, *H19*, *Matn4*, *Shisa3*, and *Clec11a* (DeSisto et al., 2020; Pietilä et al., 2023). Astrocytes were identified with *Aqp4*, *Agt*, and *Arxes2* (Dennis, Wang et al., 2024; Willis et al., 2025) and border astrocytes specifically with *Gfap* and *Myoc* (Hasel et al., 2025). Microglia were identified with *Tmem119*, *Cx3cr1*, and *Cd68* and border macrophages with *Mrc1* and *Lyz2* (Joost et al., 2019; Sankowski et al., 2024). Excitatory neurons were identified with *Plppr3*, *Rab40b*, *Smad3*, and *Bdnf* (Fuchs et al., 2022) and inhibitory neurons with *Dlx5*, *Prox1*, and *Plppr3* (upper interneurons) and *Dlx5* and *Sp9* (lower interneuron), further differentiated by restricted location (Miyoshi et al., 2015; de Lombares et al., 2019; Yao et al., 2021). OPCs and oligodendrocytes were identified with *Erbb3* and *Sox10* and OPCs specifically with *Cspg4* and *Pdgfra* (Nave and Salzer et al., 2006; Dennis, Wang et al., 2024; Willis et al., 2025)*.* VSMCs were identified with *Myh11* and *Pdgfrb* and pericytes with *Kcnj8*, *Rgs5*, and *Pdgfrb* (Vanlandewijck et al., 2018).

Each Xenium field of view (FOV) was processed and annotated individually, and Seurat SelectIntegrationFeatures, FindIntegrationAnchors, and IntegrateData functions were used to merge FOVs. After merging, Normalization, PCA, SNN analysis, clustering, and annotation were performed on merged datasets and/or subsets as described above, resulting in one merged Seurat object for each panel. The mesenchymal probeset included datasets from 6 sections/4 independent mice total, and the brain probeset analysis included 2 sections/2 independent mice plus 3 sections/3 independent mice from Willis et al. (2025) (GEO GSE266689, sections GSM8647390, GSM8647391, GSM8647392;5 sections/5 mice total).

### Cellular proximity analysis

To perform nearest neighbor analysis, we used the Giotto toolkit as described by Dries et al. (2021). We created spatial networks using both Delauney and kNN methods with a maximum distance of 400 and used the kNN method with the *cellProximityEnrichment* function to create a cell proximities frequency table. Each cell was assigned a label such as astrocyte or microglia, and we used a permutation test to compare our interaction (cell proximity count) data to a null distribution created from a random permutation of cell labels while keeping the positions fixed. Hence, the overall number of cells and proportion of each cell type are preserved but the spatial arrangement is randomized. We used 1,000 simulations to create the null distribution of expected cell proximity counts for comparison with our observed data. We used the fdr adjustment method for our *p* value adjustment. P_higher (enrichment) and p_lower (depletion; raw one-sided *p* values) assessed the probability that the observed frequency is either significantly higher (enrichment) or lower (depletion). This is shown further in the Fruchterman layout using the *cellProximityNetwork* function, where green lines indicate a depleted interaction when compared with null distribution and black and enriched interaction.

### Antibodies

We used the following primary antibodies for immunostaining; goat anti-PDGFRα (R&D Systems, catalog #AF1062; RRID: AB_2236897), rabbit anti-Laminin (Abcam, catalog #Ab11575; RRID: AB_298179), rabbit anti-CX30 (Invitrogen, catalog #71-2000; RRID AB_2533979), rabbit anti-CX26 (Invitrogen, catalog #51-2800, Lot#XA340183; RRID AB_2533903), rat anti-GFAP (Invitrogen, catalog #13-0300, Lot#WI331291; AB_2532994), rabbit anti-MATN4 (LSBio, catalog #LS-C108492-0.1, Lot#217995 RRID: RRID:AB_10637203). Fluorescently labeled highly cross-absorbed secondary antibodies were purchased from Jackson ImmunoResearch and used at a dilution of 1:300–1:500. Donkey Anti-Rat Alexa Fluor 488 (catalog #712-545-153; RRID: AB_2340684), Donkey Anti-Rabbit Alexa Fluor 555 (catalog #711-565-152; RRID: AB_3095471), Donkey Anti-Goat Alexa Fluor 647 (catalog #705-605-147, RRID: AB_2340437).

### Experimental design and statistical tests

PCA was performed using highly variable genes and UMAPs generated using the top principal components detected in the datasets. Two-dimensional t-SNE projections were generated using the top principal components (RunTSNE, Seurat). The same principal components were also used to execute SNN-Cliq-inspired clustering (FindClusters, Seurat) with iteratively increasing resolution until the number of differentially expressed genes (FindMarkers, Seurat, where *p* < 0.01 FWER, Holm method) between the most similar clusters reached ∼30 genes. We used the Pearson’s correlation coefficient test (cor.test function in R) to determine similarities between leptomeningeal and dural mesenchymal cells. For the cell proximity analysis, we used a permutation test to compare a generated random null distribution to our collected data.

### GEO accession numbers

Raw data for all of the new datasets described in this manuscript are publicly available in the GEO public repository. The accession number for the scRNA-seq datasets is GSE297616 and for the Xenium datasets is GSE296134.

## Results

### scRNA-seq identifies cell types within the meningeal:brain interface of the adult murine cerebral cortex

To ask about cells that comprise the cortical brain surface interface, we initially performed single-cell RNA sequencing (scRNA-seq) and used two different dissection protocols to obtain a global overview of the cells at this interface (see Materials and Methods). In one approach, we isolated cortical surface-associated cells, including the leptomeninges, by dissecting the top of the cortex, adhering the ventral surface to a coverslip, and then liberating single cells enzymatically (Extended Data Fig. 1-1*A*). In the second approach, we enriched for bone-associated cells such as the dura by removing the skull, dissecting the associated connective tissue, and enzymatically dissociating single cells. For both dissections, we used FACS to select for viable cells and then isolated and sequenced single cells using the 10x Genomics Chromium platform. In total, we performed four separate runs for the cortical surface and two runs for bone-associated cells, using 6–10 mice per run.

The resultant transcriptomes were analyzed using a previously described pipeline (see Materials and Methods for details). After filtering the datasets for low-quality cells with few expressed genes or high mitochondrial proportions and cell doublets, we obtained 810–2,709 single-cell transcriptomes in each of the six independent datasets (9,436 cells total). We then merged the transcriptomes from the bone-associated datasets and those from the cortical surface datasets separately. We used genes with high variance to compute principal components as inputs for projecting cells in two dimensions using UMAPs, and clustering was performed using a shared nearest neighbors-cliq (SNN-cliq)-inspired approach built into the Seurat R package at a range of resolutions (Hao et al., 2024). Since there was some segregation between the four different cortical surface datasets, we normalized the merged dataset using one iteration of Harmony batch correction (Korsunsky et al., 2019; Extended Data Fig. 1-1*B*). We then used gene expression overlays for well-validated marker genes to define cell types.

In both preparations, this analysis (Fig. 1*A*,*B*; Extended Data Fig. 1-1*B–E*) identified *Pdgfra*-positive mesenchymal cells and vasculature-associated cells including endothelial cells, pericytes, and VSMCs. We also identified peripheral immune populations including macrophages, T cells, and B cells. Since mice were not perfused prior to single-cell isolation, these could be either blood-borne or tissue-localized. Some cell types were specific to one or the other isolation. For the cortical surface analysis (Fig. 1*A*; Extended Data Fig. 1-1*A–C*), we identified CNS cell types including glial cells, microglia, a small number of neurons, and choroid plexus cells. In contrast, the bone-associated preparation (Fig. 1*B*; Extended Data Fig. 1-1*D,E*) did not include CNS cell types or microglia but did include Schwann cells derived from peripheral cranial nerves (Carr et al., 2019; Bastedo et al., 2024).

10.1523/ENEURO.0046-25.2025.f1-1 Figure 1-1 ***scRNA-seq to analyze adult murine cortical leptomeninges and dura-associated cell types*. (A)** Schematic of methods used to generate cells for the scRNA-seq datasets shown in Fig. 1A and B. The top illustrates the brain surface dissection and digestion and the bottom the bone-associated dissection. **(B)** UMAP visualization of merged transcriptomes from 4 independent cortical surface-associated (CSA) scRNA-seq runs (CSA1-4). Transcriptomes from each run are colored as per the adjacent legend. The top panel shows the cluster UMAP without Harmony batch correction, and the bottom with one iteration of Harmony. The bottom UMAP is also annotated for cell types. CP = choroid plexus, EC = endothelial cells, Lepto = leptomeninges, Ast/OPC = astrocytes and oligodendrocyte precursor cells, Neu = neurons, VSMC = vascular smooth muscle cells. **(C)** The batch-corrected, merged dataset in (B, bottom panel) was overlaid for expression of marker genes specific to different cell types. Expression levels are color-coded as per the adjacent keys. **(D)** UMAP visualization of merged transcriptomes from 2 independent bone-associated scRNA-seq runs (BA1, BA2). Transcriptomes from each run are colored as per the adjacent legend. The UMAP is also annotated for cell types. EC = endothelial cells. **(E)** The merged bone-associated dataset in (D) was overlaid for expression of marker genes specific to different cell types. Expression levels are color-coded as per the adjacent keys. **(F, G)** *Pdgfrb*-positive mesenchymal cells were subsetted from the bone-associated and cortical surface-associated datasets shown in (B) and (D), and reanalyzed. The annotated UMAPs (upper left panels) show the subsetted transcriptomes colored by cluster, and annotated for *Pdgfra*-positive dural or leptomeningeal cells (Dural in F, Lepto in G), and for *Pdgfra*-negative pericytes and vascular smooth mucle cells (VSMCs). The remainder of the panels show expression overlays for genes characteristic of each of the different mesenchymal cell types. Expression levels are color-coded as per the adjacent keys. **(H)** Pearson correlation analysis of averaged expression of each detected gene in leptomeningeal (y-axis) versus dural (x-axis) cell transcriptomes from the merged dataset shown in Fig. 1E. **(I)** Violin plots showing relative expression levels of select mRNAs that were identified in the differential gene expression analysis as being highly-enriched in leptomeningeal (colored blue) versus dural (colored orange) cells, respectively. Dots represent expression levels in individual cells. Download Figure 1-1, TIF file.

**Figure 2.**
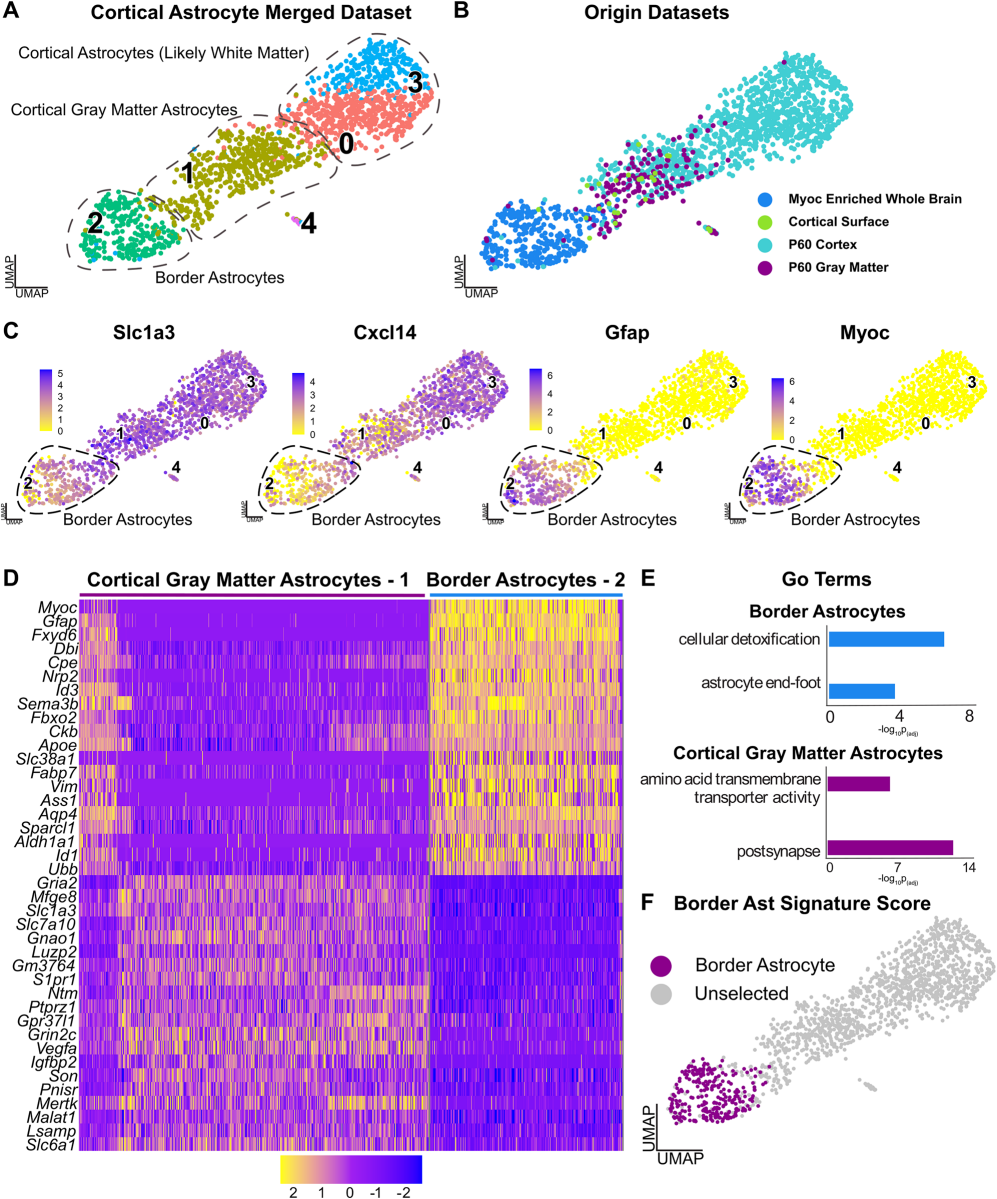
Identification of transcriptionally distinct border astrocytes by scRNA-seq analysis. Also see Extended Data Figures 2-1, 2-2, and 2-3. ***A***, ***B***, Astrocyte transcriptomes were subsetted from four different murine scRNA-seq datasets: cortical surface-associated cells as in Figure 1*A*, P60 whole cortex and P60 cortical gray matter from Dennis et al. (2024) (GEO GSE255405; Extended Data Fig. 2-1*A,B*), and whole-brain astrocytes from Hasel et al. (2021) (GEO GSE148611). In the latter case, only astrocytes enriched for *Myoc* and *Gfap* were extracted (Extended Data Fig. 2-1*C,D*). These astrocyte transcriptomes were merged and batch corrected using one iteration of Harmony. The resultant merged dataset is shown as UMAP visualizations with the transcriptionally distinct clusters numbered and color coded in ***A*** and datasets of origin shown in ***B***. ***C***, UMAPs as in ***A*** and ***B*** overlaid for expression of two pan-astrocyte mRNAs, *Slc1a3* and *Cxcl14*, and two brain interface-enriched mRNAs, *Myoc* and *Gfap*. The putative border astrocytes are circled. Expression levels are color coded as per the adjacent keys. ***D***, Single-cell heatmap of select mRNAs that were differentially expressed (Extended Data Fig. 2-2) in the comparison between the putative border astrocytes (cluster 2 in panel ***A***) and cortical gray matter astrocytes (cluster 1 in panel ***A***). Every row represents expression in an individual cell, and levels are color coded as per the adjacent key. ***E***, Gene ontology was performed on the differentially expressed genes identified in the comparison between border astrocytes and cortical gray matter astrocytes (>1.3-fold average change, *p* adj < 0.05; Extended Data Figs. 2-2, 2-3). Shown are selected categories (*y*-axis) and the adjusted *p* value (*x*-axis). ***F***, A transcriptional signature for the putative border astrocytes (cluster 2 in ***A***) was defined using the 20 most differentially expressed mRNAs in the border versus cortical gray matter astrocyte comparison (Extended Data Fig. 2-2). Shown is a UMAP as in ***A*** with cells expressing a border astrocyte transcriptional signature score >0.5 highlighted in purple.

10.1523/ENEURO.0046-25.2025.f1-2 Figure 1-2 ***Genes differentially expressed in leptomeningeal and dural mesenchymal cells.*** Differential gene expression analysis was performed on the merged *Pdgfra*-positive leptomeningeal and dural clusters shown in Fig. 1E. Differentially expressed genes were defined as those expressed in >10% of cells in either group with a Bonferroni adjusted p-value < 0.05 (Adj. p-value) and ≥ 1.3 average log2 fold change (Avg log2FC). *Denotes genes included in the dural cell signature. **Denotes genes included in the leptomeningeal cell signature. Download Figure 1-2, XLS file.

**Figure 3.**
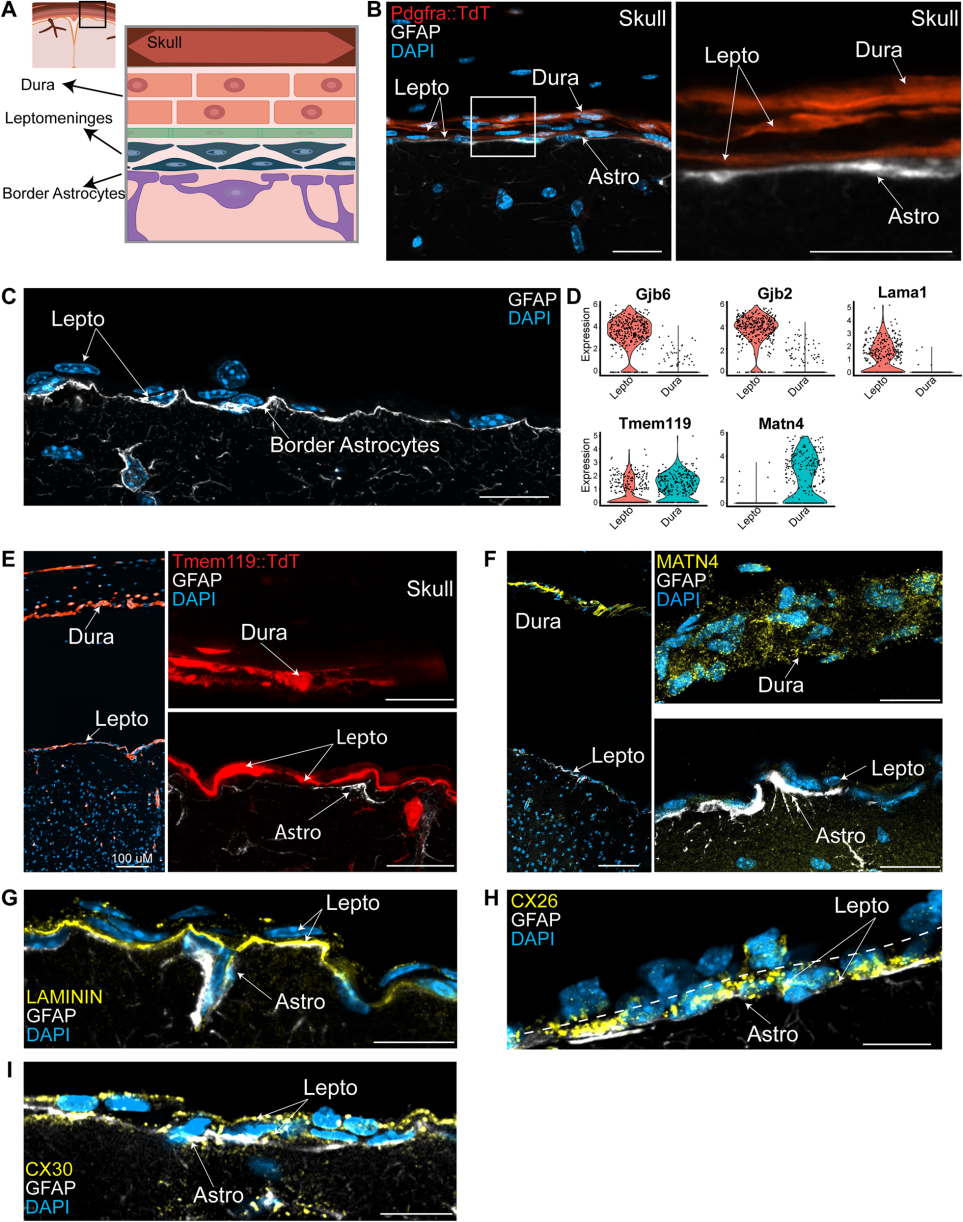
Lineage tracing and immunostaining identify the leptomeninges and the dura at the cortical brain interface. Also see Extended Data Figure 3-1. ***A***, Schematic showing the region of the cortex and overlying skull that were analyzed in the lineage tracing and immunostaining studies. ***B***, Ten-week-old *Pdgfra-CreERT2;R26RtdT* mice were treated with tamoxifen and tissues collected 10 d after the final administration. The brain and skull were decalcified, and sagittal cortical sections were immunostained for GFAP (white) and counterstained with DAPI (blue). Shown is a representative confocal image of the brain interface and skull showing TdTomato (red), GFAP (white), and DAPI (blue). The boxed region is shown at higher magnification to the right without the DAPI. Lepto, leptomeninges; Astro, border astrocytes. ***C***, Representative confocal image of a sagittal wild-type mouse brain interface section prepared as in ***B***, immunostained for GFAP (white) and counterstained with DAPI (blue). Lepto, leptomeninges. ***D***, Violin plots showing the relative expression of *Gjb6*, *Gjb2*, *Lama1*, *Tmem119*, and *Matn4* in the leptomeningeal (colored red) versus dural (colored turquoise) cells shown in Figure 1*E*,*F*. Dots represent individual transcriptomes. ***E***, Ten-week-old *Tmem119CreErt2;R26RtdT* mice were treated with tamoxifen and tissues collected 10 d later. The brain and skull were decalcified and sagittal cortical sections immunostained for GFAP (white) and counterstained with DAPI (blue). The left panel shows a low magnification image of the skull, dura, and cortical interface showing TdTomato (red) and DAPI (blue). The space between the leptomeninges and dura is a processing artifact. The two right panels are high magnification confocal images of the same section showing TdTomato (red) and GFAP immunostaining (white) in the dura and skull (top right) and the brain interface (bottom right). Both top and bottom right panels come from the same field of view. Lepto, leptomeninges; Astro, border astrocytes. ***F***, Representative images of sagittal sections through the decalcified skull and cortex, immunostained for GFAP (white) and MATN4 (yellow) and counterstained with DAPI (blue). The left panel shows a low magnification image of the skull, dura and cortical interface. The space between the leptomeninges and dura is a processing artifact. The two right panels are high magnification confocal images of a similar section from the same brain showing MATN4 (yellow) and GFAP (white) immunostaining in the dura and skull (top right) and the brain interface including the leptomeninges (bottom right). Both top and bottom right panels come from the same field of view. Lepto, leptomeninges; Astro, border astrocytes. ***G***, High magnification confocal image of a sagittal adult cortical interface section immunostained for GFAP (white) and Laminin (yellow) and counterstained with DAPI. Note that the Laminin immunoreactivity is localized between the border astrocytes and the leptomeningeal cells. Lepto, leptomeninges; Astro, border astrocytes. ***H***, High magnification confocal image of a sagittal adult cortical interface immunostained for GFAP (white) and CX26 (yellow) and counterstained with DAPI. The hatched line delineates the border between the leptomeninges and the dura. Lepto, leptomeninges; Astro, border astrocytes. ***I***, High magnification confocal image of a sagittal adult cortical interface section immunostained for GFAP (white) and CX30 (yellow) and counterstained with DAPI. Lepto, leptomeninges; Astro, border astrocytes. For all panels, scale bars are 10 µm except for the leftmost images in ***E*** and ***F***, where scale bars are 100 μm.

10.1523/ENEURO.0046-25.2025.f1-3 Figure 1-3 ***Gene ontology for genes differentially enriched in leptomeningeal and dural cells as shown in Figure 1-2.*** g:Profiler was used to identify gene ontology terms that were overrepresented in the differentially enriched leptomeningeal versus dural mRNAs shown in Figure 1-2. Enriched terms from the Molecular Function (MF), Biological Process (BioP), and Cell Components (CC) categories are all included. Download Figure 1-3, XLS file.

We observed several distinct types of *Pdgfrb*-positive cells within these datasets. In both cortical surface- and bone-associated datasets, there were *Pdgfrb*-positive pericytes and VSMCs that were negative for *Pdgfra* and positive for either the pericyte markers *Rgs5* and *Kcnj8* or the VSMC markers *Myh11* and *Acta2* (Fig. 1*C*,*D*; Extended Data Fig. 1-1*F,G*). These vasculature mural cells are likely associated with meningeal blood vessels and parenchymal vasculature (Vanlandewijck et al., 2018). More importantly for our analysis, we also identified cells positive for *Pdgfrb*, *Pdgfra*, and *Col1a1* that included putative dural meningeal cells in the case of the bone-associated dataset and leptomeningeal cells in the case of the cortical surface-associated dataset (Fig. 1*C*,*D*; Extended Data Fig. 1-1*F,G*). Since there is increasing evidence that the family of cells expressing these mRNAs, which includes fibroblasts, are heterogeneous (Carr et al., 2019; Storer et al., 2020; Li et al., 2024), then from hereon we have called these dural cells, leptomeningeal cells, or when their precise identity or location is unknown, *Pdgfra*-positive mesenchymal cells. Thus, our findings validated the dissections and identified likely meningeal mesenchymal cells (Vanlandewijck et al., 2018; DeSisto et al., 2020; Pietilä et al., 2023).

### Dural meninges and leptomeninges have distinct transcriptional signatures

We further analyzed the potential meningeal cells by subsetting, merging, and reanalyzing *Pdgfra*-positive cells from the bone-associated and cortical surface-associated datasets. We performed one iteration of Harmony batch correction to ensure integration without overcorrection (Korsunsky et al., 2019). UMAP-based cluster visualization (Fig. 1*E*) and analysis of the dataset of origin (Fig. 1*F*) identified two major clusters of *Pdgfra*-positive mesenchymal cells. Cluster 1 included cells almost exclusively from the bone-associated (BA) dataset, while cluster 0 included cells predominantly from the cortical surface-associated (CSA) dataset. We interpret these as bone-associated dural cells and cortical surface-associated leptomeningeal cells, respectively.

We directly compared these two populations using several approaches. First, we performed Pearson’s correlation analysis of averaged gene expression. The two mesenchymal cell populations were quite distinct with *r* = 0.844 (Extended Data Fig. 1-1*H*). Second, we performed differential gene expression analysis (Extended Data Fig. 1-2) and identified 1,239 differentially expressed genes (average log2 fold change ≥1.3, adj *p* value <0.5, expression in at least 10% of cells). A total of 872 genes were enriched in the putative dural cells and 367 in the putative leptomeningeal cells. GO analysis (Kolberg et al., 2023; Fig. 1*G*; Extended Data Fig. 1-3) showed that the dural cells were enriched for terms like extracellular matrix organization, skeletal system development, and ossification, consistent with their role as skull stromal cells. In contrast, the leptomeningeal cells were enriched for terms like active transmembrane transporter activity, cell junction organization, and cell–cell signaling, consistent with their role as brain interface cells. Heatmaps and violin plots (Fig. 1*H*,*I*; Extended Data Fig. 1-1*I*) confirmed enrichment for these mRNAs. The leptomeningeal cells were highly enriched for expression of the transmembrane transporter *Slc22a6*, the basal lamina component *Lama1*, the connexins *Gjb2* and *Gjb6*, and the prostaglandin D2 synthase *Ptgds*, which is associated with cerebral spinal fluid leak (Kondabolu et al., 2011). Conversely, the dural cells were enriched for *Igfbp7*, *Clec11a*, *Alpl*, *Ostn*, *H19*, and *Mgp*, all of which are implicated in osteochondrogenesis (Zhang et al., 2018; Sato et al., 2021; Liu et al., 2023). We used the top 50 differentially expressed dural versus leptomeningeal genes to define transcriptional signatures that readily distinguished these two populations (Extended Data Fig. 1-2). The leptomeningeal signature (Fig. 1*J*, blue) included genes such as *Ptgds*, *Slc22a6*, *Gjb6*, *Gjb2*, *Bmp6*, and *Lama1*, while the dural signature (Fig. 1*J*, red) included genes such as *Matn4*, *Igfbp7*, *H19*, *Clec11a*, and *Smoc2*. Thus, both dura and leptomeninges are composed of *Pdgfra*-positive mesenchymal cells, but the dural cells are more analogous to bone stromal cells while the leptomeningeal cells have a transcriptional profile consistent with a brain interface role.

### Brain interface astrocytes are transcriptionally distinct from cortical gray matter astrocytes

The brain interface is composed of leptomeningeal cells and closely associated CNS astrocytes. We therefore asked if the brain interface-associated astrocytes were also a transcriptionally distinct population using scRNA-seq. To do so, we examined the cortical surface scRNA-seq dataset. Since this contained relatively few astrocyte transcriptomes, we merged it with several additional astrocyte scRNA-seq datasets. First, we extracted cortical astrocyte transcriptomes from two recently published scRNA-seq datasets, one including total postnatal day 60 (P60) adult cortex cells and the other P60 cortical gray matter cells (GEO GSM8072112; GSM8072111; Dennis et al., 2024; Extended Data Fig. 2-1*A,B*). Second, we took advantage of the recent finding that astrocytes of the glia limitans superficialis are enriched for *Myoc* and *Gfap* mRNAs (Hasel et al., 2025). We therefore subsetted and included *Myoc*-expressing, *Gfap*-enriched astrocytes from a previously published total brain astrocyte scRNA-seq dataset (Hasel et al., 2021; GEO GSE148611; Extended Data Fig. 2-1*C,D*). We merged all of these astrocyte transcriptomes with the astrocyte transcriptomes we had obtained in the cortical surface analysis (the dataset in Fig. 1*A*) and performed batch correction using Harmony.

This analysis (Fig. 2*A*,*B*) identified four astrocyte clusters in three distinct groups. One group of clusters (0 and 3) likely include cortical white matter astrocytes, since analysis of the dataset of origin showed they were almost completely composed of astrocytes from the total cortex dataset and did not include gray matter transcriptomes. The gray matter transcriptomes were instead in cluster 1, which also included transcriptomes from the total cortex and thus likely includes cortical parenchymal astrocytes. The final cluster (2) was largely composed of *Myoc*-enriched whole-brain astrocytes together with cells from the cortical surface dataset. All cells expressed pan-astrocyte markers such as *Slc1a3* (GLAST) and *Cxcl14*, and, as predicted, cluster 2 was enriched for *Myoc* and *Gfap* (Fig. 2*C*; Batiuk et al., 2020). From hereon, we have called these cluster 2 cells putative border astrocytes, since *Myoc* and *Gfap* are enriched in astrocytes at the brain surface.

10.1523/ENEURO.0046-25.2025.f2-1 Figure 2-1 ***scRNA-seq to analyze adult cortical and border astrocytes.* (A, B)** UMAP visualizations of transcriptomes from previously-published (Dennis et al., 2024; GEO GSE255405) scRNA-seq datasets of dissected postnatal day 60 (P60) cortical grey matter (A) or total cortex (B) tissue. Transcriptomes were reanalyzed and cell types were identified using well-characterized marker genes. The transcriptionally-distinct clusters are color-coded, and different cell types annotated and denoted by the hatched lines. **(C)** UMAP visualization of transcriptomes from a previously-published (Hasel et al., 2021; GEO GSE148611) whole brain astrocyte scRNA-seq dataset. Transcriptomes were reanalyzed and transcriptionally-distinct clusters are color-coded. The cluster most enriched for *Myoc* and *Gfap* expression (outlined) was subsetted and used for subsequent analyses. **(D)** Violin plots showing relative expression levels of *Myoc* and *Gfap* mRNAs in the putative whole brain border astrocytes (BorderAst) from the cluster outlined in (C), versus all other astrocytes (Ast) from the dataset shown in (C). Download Figure 2-1, TIF file.

10.1523/ENEURO.0046-25.2025.f2-2 Figure 2-2 ***Genes differentially expressed in border astrocytes versus cortical grey matter astrocytes.*** Differential gene expression analysis was performed on the merged astrocyte dataset shown in Fig. 2A, comparing the border astrocytes (cluster 2) and the cortical grey matter astrocytes (cluster 1). Differentially expressed genes were defined as those expressed in >10% of cells in either group with a Bonferroni adjusted p-value < 0.05 (Adj. p-value) and ≥ 1.3 average log2 fold change (Avg log2FC). *Denotes genes included in the border astrocyte gene signature. Download Figure 2-2, XLS file.

10.1523/ENEURO.0046-25.2025.f2-3 Figure 2-3 Gene ontology for genes differentially enriched in border astrocytes versus cortical grey matter astrocytes as shown in Figure 2-2. g:Profiler was used to identify gene ontology terms that were overrepresented in the differentially enriched border astrocyte versus cortical grey matter astrocyte mRNAs shown in Figure 2-2. Enriched terms from the Molecular Function (MF), Biological Process (BioP), and Cell Components (CC) categories are all included. Download Figure 2-3, XLS file.

To better understand the putative cluster 2 border astrocytes, we directly compared them with cluster 1 cortical parenchymal/gray matter astrocytes using differential gene expression analysis. A total of 575 mRNAs differed significantly between the two groups (average log2 fold change ≥1.3, adj *p* value <0.5, expression in at least 10% of cells; Extended Data Fig. 2-2). Cluster 2 border astrocytes were enriched for 381 mRNAs including *Myoc*, *Gfap*, *Fbxo2*, *Id1*, *Vim*, and *Agt*, as illustrated in a heatmap (Fig. 2*D*; Extended Data Fig. 2-2). Gene ontology analysis of these differentially enriched border astrocyte genes (Extended Data Fig. 2-3) identified terms that included cellular detoxification and astrocyte end foot (Fig. 2*E*). In contrast, cluster 1 gray matter astrocytes were enriched for 194 mRNAs, including *Gria2*, *Igfbp2*, and *Slc1a3* (Fig. 2*D*; Extended Data Fig. 2-2) and gene ontology identified terms such as postsynapse and amino acid transmembrane transporter (Fig. 2*E*; Extended Data Fig. 2-3). We used the 20 most differentially expressed genes to define a specific transcriptional signature for the putative *Myoc*-positive border astrocytes that included *Myoc*, *Gfap*, *Fbxo2*, *Slc38a1*, and *Fxyd6* (Fig. 2*F*; Extended Data Fig. 2-2).

### Visualization of leptomeningeal:astrocyte interactions at the cortical brain interface

One caveat of the scRNA-seq data is that it does not provide spatial information about the interface cells. We therefore performed two complementary morphological studies, lineage tracing plus immunostaining and single-cell spatial transcriptomics. For the lineage tracing analysis, we initially used a well-characterized mouse line where CreERT2 is driven from the endogenous *Pdgfra* locus (Chung et al., 2018). We crossed these to another mouse line carrying a floxed TdTomato transgene in the *Rosa26* locus (Madisen et al., 2010); when these crossed *Pdgfra-CreERT2;R26RtdT* mice are exposed to tamoxifen, TdTomato will be expressed in and specifically tag *Pdgfra*-positive mesenchymal cells. *Pdgfra*-positive OPCs will also be genetically tagged, but these are readily distinguished by their location, morphology, and transcriptional profiles. We therefore exposed crossed mice to tamoxifen for 5 d, isolated the cortex and overlying skull 10 d later, decalcified the skull, and analyzed sagittal sections from layer 1 through to the top of the skull (Fig. 3*A*). This analysis (Fig. 3*B*) showed that as predicted both the dura and the leptomeninges were TdTomato-positive and that the leptomeninges were only two to three layers thick in this region. Moreover, immunostaining for GFAP (Fig. 3*B*,*C*) confirmed that it was highly enriched in the border astrocytes (Fig. 2*C*) and demonstrated that the TdTomato-positive leptomeningeal cells were immediately adjacent to GFAP-positive border astrocytes. Similar findings were made by immunostaining sections for PDGFRα protein and GFAP (Extended Data Fig. 3-1*A*).

10.1523/ENEURO.0046-25.2025.f3-1 Figure 3-1 ***Characterization of the cortical brain interface by immunostaining.* (A)** High magnification confocal images of a sagittal section through the brain interface and skull, immunostained for PDGFRα (yellow) and GFAP (white), and counterstained with DAPI (blue). The left panel shows the merge and the right two immunostaining for PDGFRα or GFAP separately. Lepto = leptomeninges, Astro = border astrocytes. Scale bar = 10 μm. **(B, C)** The border astrocyte transcriptomes from cluster 2 in Fig. 2A and the leptomeningeal and dural cell transcriptomes from Fig. 1E were merged and reanalyzed. Shown are UMAPs with the clusters color-coded and annotated (B) or the datasets of origin indicated as per the adjacent color legend (C). CSA = cortical surface-associated dataset, BA = bone-associated dataset. **(D)** Gene expression overlays of the merged astrocyte plus meningeal mesenchymal cell dataset shown in (B, C) for selected genes that distinguish the different cell types. Expression levels are color-coded as per the adjacent keys. Download Figure 3-1, TIF file.

We performed a similar analysis with a *Tmem119-CreERT2;R26RtdT* mouse line that has been widely used to label microglia (Kaiser and Feng, 2019), based upon our finding that *Tmem119* is expressed in both the dura and the leptomeninges (Fig. 3*D*). As predicted, following tamoxifen treatment both the dura and leptomeninges were TdTomato-positive in the *Tmem119-CreERT2;R26RtdT* mice as were microglia located in the cortical parenchyma (Fig. 3*E*). As seen with the *Pdgfra-CreERT2*-based lineage tracing, the leptomeninges were only several cell layers thick at this point above the cortex. Moreover, immunostaining for GFAP showed that the GFAP-positive border astrocytes were closely juxtaposed to the TdTomato-positive leptomeningeal cells (Fig. 3*E*).

Both the dura and leptomeninges express *Pdgfra* and *Tmem119*, and thus these lineage tracing approaches do not distinguish the two compartments. We therefore immunostained for proteins predicted to be differentially expressed in the transcriptional data. For the dura, we immunostained decalcified sagittal cortical sections that included the skull for MATN4, which is highly enriched in the putative dural cells (Fig. 3*D*). For the leptomeninges, we immunostained similar sections for Laminin (*Lama1*), or the gap junction proteins Connexin 26 (*Gjb2*) or Connexin 30 (*Gjb6*), all of which are highly enriched in the leptomeningeal cells (Fig. 3*D*; DeSisto et al., 2020; Pietilä et al., 2023). Of these, *Gjb2* is also expressed at low levels in border astrocytes and *Gjb6* is widespread in astrocytes (Extended Data Fig. 3-1*B–D*). In all cases, we double-labeled sections for GFAP to visualize the border astrocytes. As predicted, immunoreactivity for the extracellular matrix protein MATN4 was not detectable in the leptomeninges but was observed in the dura (Fig. 3*F*). In contrast, Laminin, CX26/Gjb2, and CX30/Gjb6 were detectable in the leptomeninges but not the dura (Fig. 3*G–I*). Laminin immunoreactivity was specifically associated with the basal lamina between the GFAP-positive border astrocytes and leptomeningeal cells, while CX26 and CX30 immunoreactivity were present in a punctate pattern in the leptomeningeal cells immediately adjacent to GFAP-positive border astrocytes. As predicted (Extended Data Fig. 3-1*B–D*), CX30 mRNA was also detected in border astrocytes, as was CX26 at apparently lower levels (Lynn et al., 2011; Nagy et al., 2011; Huang et al., 2021). These studies thus identify lineage tracers for the leptomeninges, validate our findings from the scRNA-seq datasets, and highlight the close interactions between border astrocytes and leptomeningeal cells at the cortical brain interface.

### Single-cell spatial transcriptomics defines the brain interface as closely associated leptomeningeal cells, border astrocytes, and border macrophages

The lineage tracing and immunostaining studies are limited by their ability to only analyze several marker genes/proteins at a time. To obtain a more global overview of the cortical brain interface, we performed single-cell multiplexed in situ gene expression analysis using the Xenium platform (Janesick et al., 2023; Willis et al., 2025). We did this with two different probesets, one a custom 480 gene probeset optimized to distinguish different mesenchymal cell populations, including those of the meninges, and the second a standard Xenium brain probeset with a custom 100 gene add-on composed of probes allowing better resolution of non-neural brain cell types (see Materials and Methods; Extended Data Fig. 4-3).

**Figure 4.**
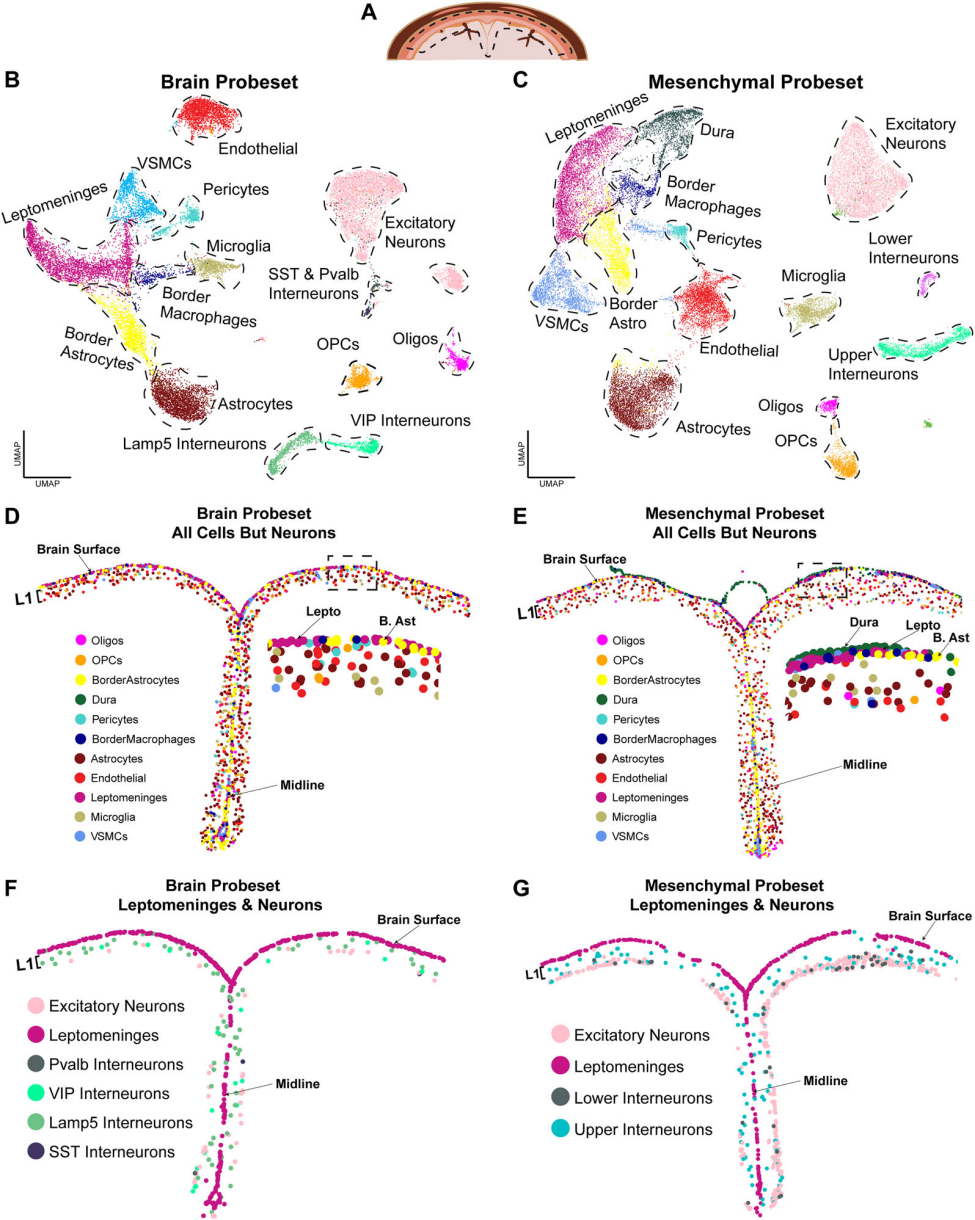
Single-cell multiplexed in situ gene expression analysis with two different probesets identifies the spatial location of cortical interface and layer 1 cell types. Also see Extended Data Figures 4-1, 4-2, and 4-3. Coronal adult mouse cortex sections at the levels shown in Extended Data Fig. 4-1*A,B* were analyzed by Xenium-based single-cell multiplexed in situ gene expression analysis with either a brain-targeted probeset targeting 347 genes or a mesenchymal probeset targeting 480 genes (Extended Data Fig. 4-3). The ROI that was analyzed is shown in ***A***. Datasets from different sections and conditions were then merged and cell types identified by marker gene expression. ***A***, Schematic of a coronal section through the adult cortex in the midline region showing the ROI that was analyzed (outlined with hatched black lines). ***B***, ***C***, UMAP cluster visualization of the merged transcriptomes resulting from analysis with the brain probeset (***B***) or the mesenchymal probeset (***C***), annotated for cell types. Each dot represents a single cell. VSMC, vascular smooth muscle cell; Oligos, oligodendrocytes; SST, somatostatin; Pvalb, parvalbumin; VIP, vasoactive intestinal peptide. ***D***, ***E***, Spatial plots of the midline and adjacent cortical interface and layer 1 from the ROI of representative sections analyzed using the brain (***D***) or mesenchymal (***E***) probesets, showing all cell types except neurons, color coded as per the legend. The boxed regions are also shown in enlarged views. L1 denotes cortical layer 1. Lepto, leptomeninges; B. Ast, border astrocytes. ***F***, ***G***, Spatial plots as in ***D*** and ***E*** from the same representative sections analyzed using the brain (***F***) or mesenchymal (***G***) probesets showing the leptomeninges and all of the neurons that were identified, color coded. L1 denotes cortical layer 1. Low magnification spatial plots in ***D–G*** were generated using Seurat with ***D*** and ***F*** showing the same ROI and ***E*** and ***G*** the same ROI. Each dot represents the centroid of one cell.

We used these probesets to analyze the leptomeninges and the adjacent cortical layer 1 in coronal forebrain sections from freshly frozen adult murine brain tissue (Fig. 4*A*; Extended Data Fig. 4-1*A,B*). For sections analyzed using the mesenchymal probeset, we also included the dura. We then performed Xenium-based multiplexed in situ gene expression analysis as described previously (Dennis et al., 2024; Willis et al., 2025) using one of the two probesets. For the mesenchymal probeset, we analyzed six coronal rostral forebrain sections at the level of the lateral ventricles from four different mice (Extended Data Fig. 4-1*A*). For the brain probeset, we ran two rostral coronal forebrain sections, one each from two different adult mouse brains (as in Extended Data Fig. 4-1*A*). We augmented this with a recently published Xenium dataset obtained using the same brain probeset to analyze three somewhat more caudal coronal sections of the adult cortex from three different mice (Willis et al., 2025; GEO GSE266689, sections GSM8647390, GSM8647391, GSM8647392; Extended Data Fig. 4-1*B*). In all cases we defined an ROI that spanned the midline and included cortical layer 1, the top of adjacent layer 2 plus the overlying leptomeninges (Fig. 4*A*). After initial processing of data from individual sections, we excluded cells where the detected genes and total transcript counts per cell were ±2.5 or more standard deviations from the mean in either parameter since these were likely cellular fragments or cellular doublets. We then merged cells from different sections/animals that had been analyzed using the same probeset.

10.1523/ENEURO.0046-25.2025.f4-1 Figure 4-1 Analysis of the cortical interface and layer one cells using single cell multiplexed in situ gene expression analysis with the brain probeset. (A, B) Coronal cortical sections were analyzed by Xenium-based single cell multiplexed *in situ* gene expression analysis. (A) shows a representative Xenium Explorer image of a rostral brain section at the level used for all mesenchymal probeset analyses and for 2 of the 5 brains analyzed with the brain probeset. (B) shows a representative Xenium Explorer image of the more caudal brain sections used to generate the brain probeset data previously published in Willis et al. (2025; GEO GSE266689, sections GSM8647390, GSM8647391, GSM8647392). The ROI that was analyzed for all sections is shown in Fig. 4A, and the annotated UMAP cluster visualizations of the resultant merged transcriptomes are shown in Fig. 4B and C. **(C)** UMAPs of the merged brain probeset data showing either the annotated clusters (bottom; the same UMAP as in Fig. 4B) or the section of origin (top) for each of the transcriptomes. Brain1-5 denotes 5 sections from 5 different mice. Each dot represents a single transcriptome. VSMCs = vascular smooth muscle cells, Oligos = oligodendrocytes, SST = somatostatin, Pvalb = parvalbumin, VIP = vasoactive intestinal peptide. **(D)** Expression overlays for selected marker genes on the UMAPs shown in (C). Expression levels are color-coded as per the adjacent keys. Space bars = 2500 μM. Download Figure 4-1, TIF file.

10.1523/ENEURO.0046-25.2025.f4-2 Figure 4-2 *Analysis of the cortical interface and layer one cells using single cell multiplexed in situ gene expression analysis.* (A, B) Coronal cortical sections as shown in Fig. 4-1A were analyzed by Xenium-based single cell multiplexed *in situ* gene expression analysis with the mesenchymal probeset. The ROI that was analyzed is shown in Fig. 4A. **(A)** UMAPs of the merged mesenchymal probeset data showing either the annotated clusters (bottom; the same UMAP as in Fig. 4C) or the section of origin (top) for each of the transcriptomes. MSC1-6 denotes 6 sections from 5 difference mice. Each dot represents a single transcriptome. VSMCs = vascular smooth muscle cells, Oligos = oligodendrocytes. **(B)** Expression overlays for selected marker genes on the UMAPs shown in (A). Expression levels are color-coded as per the adjacent keys. **(C)** Coronal adult mouse cortex sections at the level shown in Fig. 4-1B were analyzed by Xenium-based single cell multiplexed *in situ* gene expression analysis with the brain probeset. The ROI that was analyzed is shown in Fig. 4A. Shown are spatial plots of the midline and adjacent cortical interface and layer one from the ROI of a representative section illustrating all cell types except neurons (left) or leptomeninges and neurons (right), color-coded as per the legends. L1 denotes cortical layer one. Download Figure 4-2, TIF file.

10.1523/ENEURO.0046-25.2025.f4-3 Figure 4-3 ***Xenium probesets*.** Shown are the mesenchymal custom probeset, comprised of probes targeting 480 genes, as well as the custom add-on brain probeset targeting 100 genes that was used in conjunction with the 10X predesigned 247 gene mouse brain panel. The numbers of probes per gene were tuned for each Xenium custom add-on panel to ensure robust detection and isoform coverage, while avoiding optical crowding. Probe number selection was informed by the 10X Xenium panel designer. Download Figure 4-3, XLS file.

With the mesenchymal probeset, we ultimately obtained 29,644 total cellular transcriptomes from the merged ROIs of six sections/four brains and with the brain probeset, we obtained 20,371 transcriptomes from merged ROIs of one section each from five brains. With both probesets, transcriptomes from the different sections/brains were well integrated, as shown on UMAPs (Fig. 4*B*,*C*; Extended Data Fig. 4-1*C*; Extended Data Fig. 4-2*A*). With two exceptions, both probesets defined the same cell types, as determined by analysis of a panel of marker genes (see Materials and Methods; Fig. 4*B*,*C*; Extended Data Fig. 4-1*C,D*; Extended Data Fig. 4-2*A,B*). The exceptions were dural cells, which as predicted were only present in the mesenchymal probeset sections, and interneurons (discussed further below).

Spatial plots identified the locations of the different identified cell types, with similar spatial plots obtained on tissue sections from different mice (Fig. 4*D–G*; Extended Data Fig. 4-2*C*). The brain interface was composed of leptomeningeal cells, macrophages, and border astrocytes that were tightly packed and interspersed with vasculature mural cells and endothelial cells that were associated with penetrating blood vessels. Layer 1 was instead relatively cell sparse, and included astrocytes, oligodendrocytes, OPCs, and vasculature-associated cells. As predicted, the only neurons in layer 1 were interneurons (Fig. 4*F*,*G*; Extended Data Fig. 4-2*C*). The brain probeset defined these as expressing *Lamp5* or *Vip* (Fig. 4*B*,*F*; Extended Data Fig. 4-2*C*), consistent with previous immunocytochemical analyses (Schuman et al., 2019; 2021). The mesenchymal probeset instead defined two interneuron clusters that differed in their spatial locations (from hereon called upper or lower interneurons; Fig. 4*C*,*G*). The border of layer 1 was clearly delineated by layer 2 excitatory neurons and a few *Somatostatin-* or *Parvalbumin*-expressing interneurons that were included in the ROI (Fig. 4*F*,*G*; Extended Data Fig. 4-2*C*), again as previously reported (Tremblay et al., 2016; Lim et al., 2018; Yao et al., 2021).

**Figure 5.**
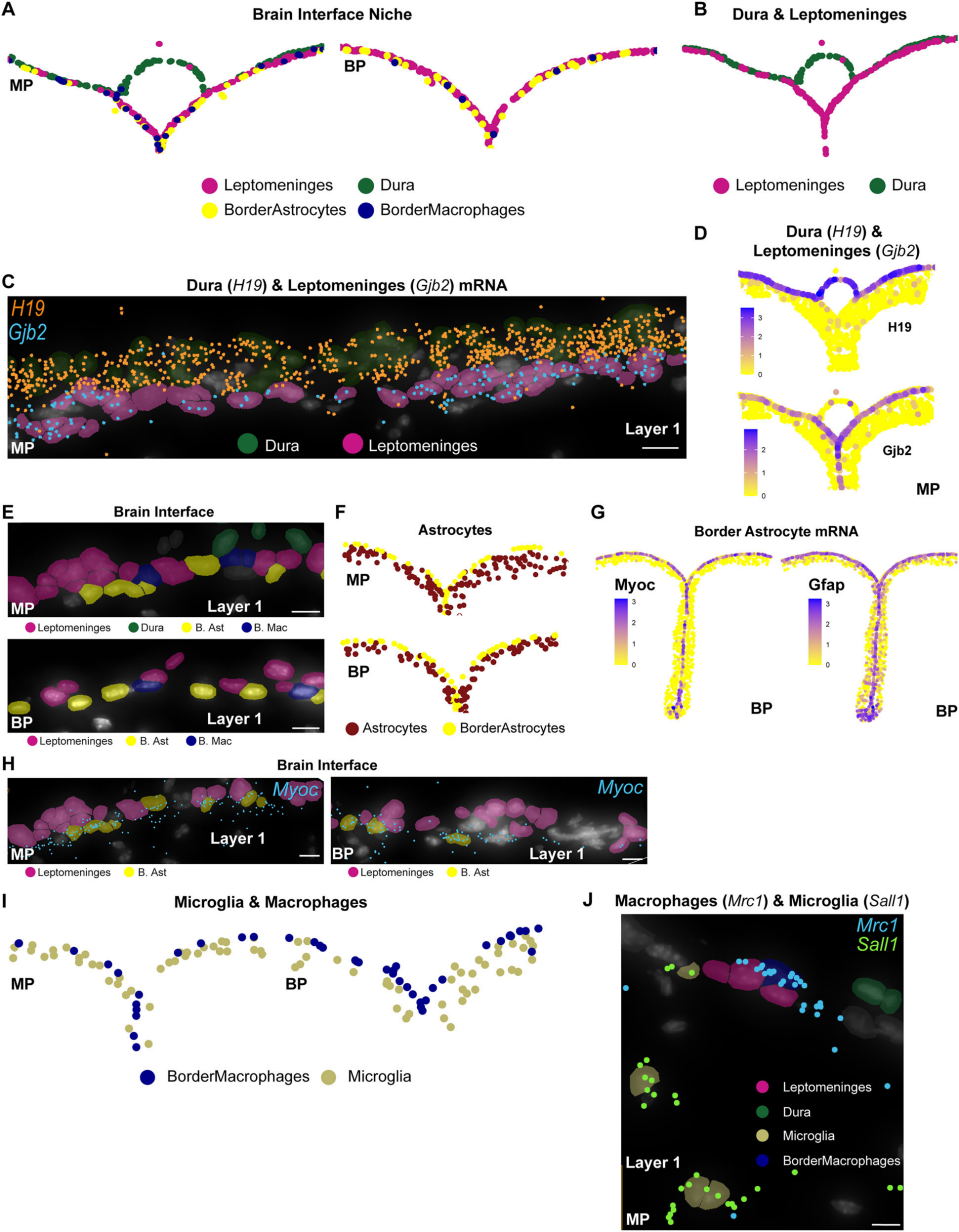
Single-cell spatial transcriptomic analysis defines the cortical brain interface structure and cell types. Also see Extended Data Figure 5-1. Coronal adult mouse cortex sections at the levels shown in Extended Data Fig. 4-1*A,B* were analyzed by Xenium-based single-cell multiplexed in situ gene expression analysis with either a brain probeset targeting 347 genes or a mesenchymal probeset targeting 480 genes (Extended Data Fig. 4-3). The ROI and UMAPs of the resultant merged datasets are shown in Figure 4*A–C*. ***A***, Spatial plots of the cortical interface analyzed with the mesenchymal (MP, left) or brain (BP, right) probesets showing leptomeningeal cells (pink), border astrocytes (yellow), border macrophages (dark blue), and dural cells (green, mesenchymal probeset only). ***B***, Spatial plot of the cortical interface and midline analyzed with the mesenchymal probeset showing dural (green) and leptomeningeal (pink) cells. ***C***, High-resolution Xenium Explorer image of the cortical interface region analyzed with the mesenchymal probeset (MP) showing expression of *H19* mRNA (orange dots) and *Gjb2* mRNA (bright blue dots) relative to the leptomeningeal cells (pink) and dural cells (green). Nuclei of other cell types (white) are also shown. ***D***, Spatial plots of the dural mRNA *H19* (top) or the leptomeningeal mRNA *Gjb2* (bottom) in all cells at the cortical interface, midline, and layer 1 analyzed using the mesenchymal probeset (MP). Relative mRNA expression levels are coded as per the adjacent keys. ***E***, High-resolution Xenium Explorer images of the cortical interface region analyzed with the mesenchymal (MP, top) or brain (BP, bottom) probesets showing the spatial arrangement of leptomeningeal cells (pink), border astrocytes (yellow), border macrophages (blue), and dural cells (green, mesenchymal probeset only). Nuclei are also shown (white/gray). ***F***, Spatial plots of the cortical interface and layer 1 analyzed with the mesenchymal (MP, top) or brain (BP, bottom) probesets showing border astrocytes (yellow) and parenchymal astrocytes (brown). ***G***, Spatial plots of *Myoc* and *Gfap* mRNA expression in all cells of the full ROI analyzed using the brain probeset (BP). Relative expression levels are coded as per the adjacent keys. ***H***, High-resolution Xenium Explorer image of the cortical interface analyzed with the mesenchymal (MP, left) or brain (BP, right) probesets showing expression of *Myoc* mRNA (light blue dots) relative to the leptomeningeal cells (pink) and border astrocytes (yellow). Nuclei of other cell types (white) are also shown. ***I***, Spatial plots of the cortical interface and layer 1 analyzed with the mesenchymal (MP, left) or brain (BP, right) probesets showing border macrophages (blue) and microglia (tan). ***J***, High-resolution Xenium Explorer image of the cortical interface and layer 1 analyzed with the mesenchymal probeset (MP) showing expression of *Mrc1* mRNA (bright blue dots) and *Sall1* mRNA (bright green dots) relative to the border macrophages (dark blue), microglia (tan), leptomeningeal cells (pink), and dural cells (darker green). Nuclei of other cell types (white) are also shown. Low magnification spatial plots in ***A***, ***B***, ***D***, ***F***, ***G***, and ***I*** were generated using Seurat and show part of the ROI centered on the cortical midline region from one representative section each, with each dot representing the centroid of one cell. High-resolution spatial plots in ***C***, ***E***, ***H***, and ***J*** were generated using Xenium Explorer. Scale bars, 10 μm.

We used these datasets to analyze the brain interface and layer 1 cellular neighborhoods in greater detail. Consistent with the immunostaining analyses (Fig. 3), the leptomeninges at the brain interface were a continuous layer of cells that were lined by border astrocytes on the CNS side (Fig. 5*A*). Resident macrophages were scattered along this interface. These are likely analogous to the previously described border macrophages (Jordão et al., 2019; Kolabas et al., 2023; Pietilä et al., 2023). The dural cells were located on the skull side of these interface cells (Fig. 5*B*), as predicted. They were transcriptionally distinct from the leptomeningeal cells, expressing mRNAs such as *Matn4* and *H19* but not the leptomeningeal mRNA *Gjb2* (Fig. 5*C*,*D*).

10.1523/ENEURO.0046-25.2025.f5-1 Figure 5-1 ***Genes differentially expressed in border macrophages versus cortical microglia.*** Differential gene expression analysis was performed on the border macrophages and microglia from the cortical surface-associated and bone-associated datasets in Fig. 1A and B. Differentially expressed genes were defined as those expressed in >10% of cells in either group with a Bonferroni adjusted p-value < 0.05 (Adj. p-value) and ≥ 1.3 average log2 fold change (Avg log2FC). Download Figure 5-1, XLS file.

A higher resolution analysis highlighted the close association between the different brain interface cell types (Fig. 5*E*). The cortical surface leptomeninges were 2–3 cells wide, with the lowest layer immediately adjacent to the border astrocytes. The border macrophages were interspersed within this leptomeningeal layer. Each of these interface-associated cell types differed transcriptionally from similar cells within other regions of the meningeal/cortical layer 1 tissue. Specifically, the border astrocytes were transcriptionally distinct and spatially segregated from their parenchymal astrocyte counterparts in layer 1 (Fig. 5*F*) and, consistent with the scRNA-seq, were enriched for *Myoc* and *Gfap* (Fig. 5*G*,*H*). The border macrophages were also transcriptionally and spatially distinct from the parenchymal microglia (Fig. 5*I*). The border macrophages were closely associated with the leptomeningeal cells, with the occasional macrophage in the parenchyma where they were associated with vasculature cells and were presumably present in blood vessels.

To confirm these macrophage/microglia cell type assignments, we performed differential gene expression (Extended Data Fig. 5-1) on the macrophages and microglia from the cortical surface-associated scRNA-seq data (Fig. 1*A*). This analysis showed that surface macrophages versus microglia were enriched for *Mrc1* versus *Sall1*, respectively, as previously reported (Jurga et al., 2020; Paolicelli et al., 2022). Since probes for both of these genes were present in the mesenchymal probeset, we analyzed their expression spatially. This analysis confirmed that the cells identified as border macrophages were positive for *Mrc1* while those identified as microglia were specifically positive for *Sall1* (Fig. 5*J*).

### Distinguishing the arachnoid and pial leptomeningeal layers

**Figure 6.**
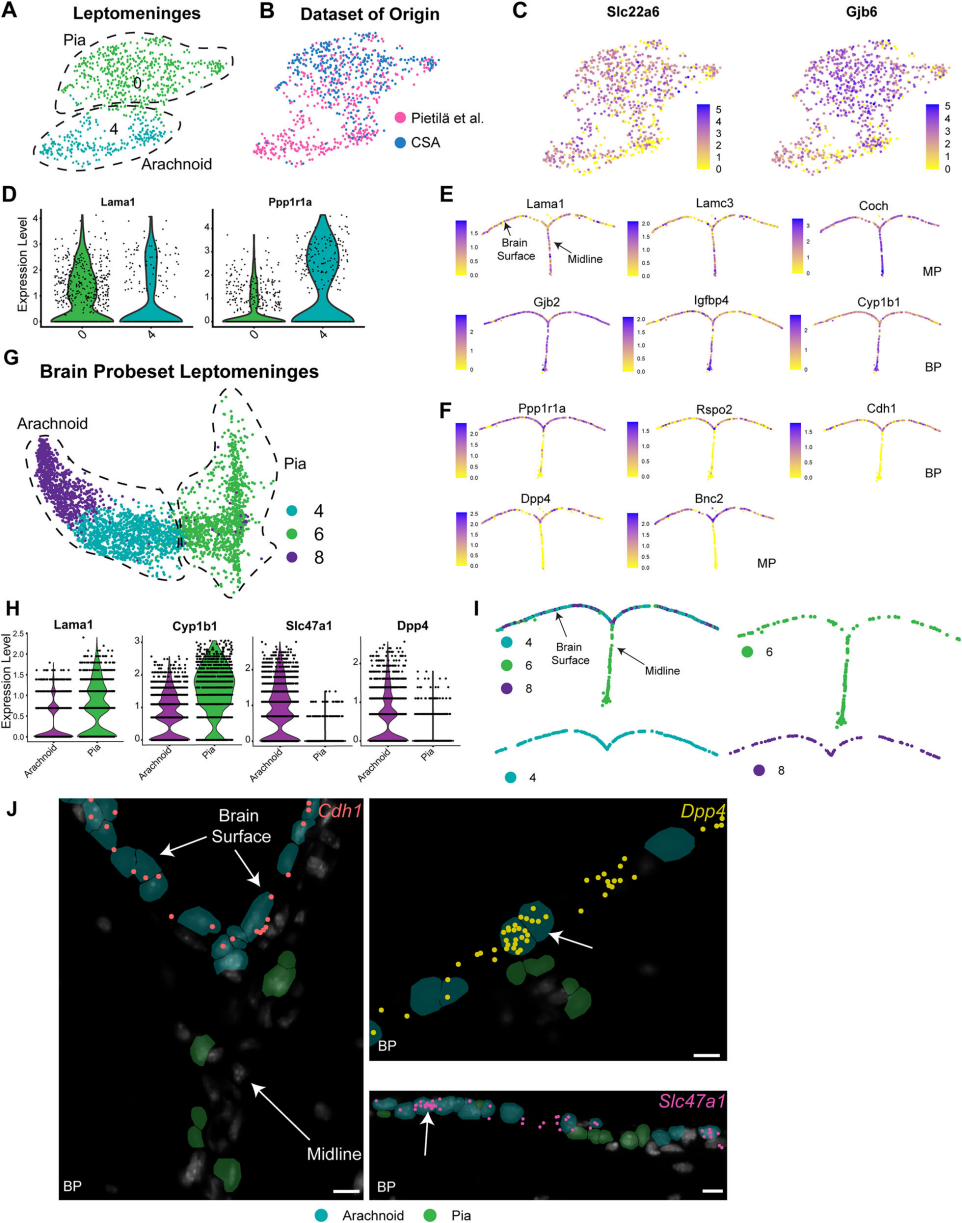
Distinguishing the arachnoid and pial leptomeningeal layers using scRNA-seq and single-cell spatial transcriptomics. Also see Extended Data Figures 6-1 and 6-2. ***A–D***, scRNA-seq-derived mesenchymal cell transcriptomes from Pietilä et al. (2023) (GEO GSE227713; shown in Extended Data Fig. 6-1*A*) were merged with the meningeal cell transcriptomes from Figure 1*E* and batch effects minimized using two iterations of Harmony. A UMAP of the total merged, annotated dataset is shown in Extended Data Figure 6-1*B*. ***A***, ***B***, UMAP visualizations of the leptomeningeal cells from the merged total meningeal cell scRNA-seq dataset (Extended Data Fig. 6-1*B*) showing the potential pial and arachnoid cell clusters (***A***) and their datasets of origin (***B***). ***C***, UMAP as in ***A***, overlaid for two pan-leptomeningeal mRNAs, *Slc22a6* and *Gjb6*. Gene expression levels are color coded as per the adjacent keys. ***D***, Violin plots showing relative expression of two mRNAs, *Lama1* and *Ppp1r1a*, in cluster 0 versus 4 (the potential pial versus arachnoid cells) in the UMAP shown in ***A***. Dots represent individual transcriptomes. ***E***, Spatial expression plots of mRNAs differentially enriched in the cluster 0 putative pial cells from ***A*** (also see Extended Data Fig. 6-2), in the leptomeningeal cells identified by Xenium using either the mesenchymal probeset (MP) or the brain probeset (BP; as shown in Fig. 4*F*,*G*). Also shown is a similar spatial plot for the pial mRNA *Lama1*. Relative mRNA expression levels are coded as per the adjacent keys. ***F***, Spatial expression plots of mRNAs differentially enriched in the cluster 4 putative arachnoid cells from ***A*** (also see Extended Data Fig. 6-2), in the leptomeningeal cells identified by Xenium using either the mesenchymal probeset (MP) or the brain probeset (BP; as shown in Fig. 4*F*,*G*). Also shown is a similar spatial plot for the arachnoid mRNA *Ppp1r1a*. Relative mRNA expression levels are coded as per the adjacent keys. ***G***, UMAP cluster visualization of the leptomeningeal transcriptomes from the Xenium brain probeset data (Fig. 4*B*), shown at higher resolution and annotated for potential pial versus arachnoid cells. The transcriptionally distinct clusters (4, 6, and 8) are color coded as per the adjacent key. ***H***, Violin plots showing relative expression levels of two pial mRNAs, *Lama1* and *Cyp1b1*, and two arachnoid mRNAs, *Slc47a1* and *Dpp4*, in the potential arachnoid versus pial leptomeningeal cells as annotated in ***G***. Purple and green denote arachnoid and pial cells, respectively. Each dot corresponds to expression in an individual cell. ***I***, Spatial plots of the cortical interface and midline showing spatial distribution of the cluster 6 pial cells (green) and the clusters 4 and 8 arachnoid cells (turquoise and purple, respectively), as annotated in ***G***. Note that the pial but not arachnoid cells extend down the midline. ***J***, High-resolution Xenium Explorer image of the cortical interface (all panels) and midline (left panel) analyzed with the brain probeset (BP) showing expression of the arachnoid mRNAs *Cdh1* (red dots), *Dpp4* (yellow dots), and *Slc47a1* (pink dots) relative to the arachnoid and pial cells (turquoise and green, respectively) as annotated in ***G***. Nuclei of other cell types (white) are also shown. Note that the pial cells extend down the midline (left panel). Low magnification spatial plots in ***E***, ***F***, and ***I*** were generated using Seurat, and each dot represents the centroid of one cell. High-resolution spatial plots in ***J*** were generated using Xenium Explorer. Scale bars, 10 μm.

These analyses identified the leptomeninges but did not distinguish arachnoid and pial cells. To do this, we took advantage of two previously published brain surface scRNA-seq datasets characterizing various meningeal populations (Pietilä et al., 2023; GEO GSE227713; see Materials and Methods for details). We ran these datasets through our pipeline, identified *Pdgfra*-positive mesenchymal cell transcriptomes, and then subsetted and reanalyzed these on their own. This analysis identified several distinct meningeal mesenchymal cell populations as previously described (Extended Data Fig. 6-1*A*). We merged these with the dural and leptomeningeal cell subset described here (from Fig. 1*E*) and used two iterations of Harmony to correct for differences in read-depth (Extended Data Fig. 6-1*B–D*). This analysis identified two leptomeningeal cell clusters (0 and 4) expressing *Slc22a6* and *Gjb6* and two mRNAs expressed in the developing leptomeninges, *Crabp2* and *Aldh1a2* (Fig. 6*A–C*; Extended Data Fig. 6-1*E*). Both clusters included intermingled cells from the cortical brain surface and Pietilä et al. datasets (Fig. 6*A*,*B*). Notably, cluster 0 was enriched for *Lama1* and cluster 4 for *Ppp1r1a* (Fig. 6*D*), identifying them as potential pial versus arachnoid cells, respectively.

10.1523/ENEURO.0046-25.2025.f6-1 Figure 6-1 ***Spatially defining arachnoid and pial cells at the cortical brain interface*. (A)** Two adult mouse brain surface scRNA-seq datasets from Pietilä et al. (2023; GEO GSE227713) were run through our pipeline, and analyzed and annotated as in the original publication (see Materials and Methods). Shown is the resultant cluster UMAP and annotations, with BFB4 and BFB5 corresponding to brain fibroblast1 and brain fibroblast5. The clusters labelled leptomeninges correspond to BFB1-3 in Pietilä et al. (2023; Fig. 1D in that paper). **(B, C)** The transcriptomes shown in (A) were merged with the leptomeningeal and dural cell transcriptomes from Fig. 1E, run through our pipeline, and two rounds of Harmony batch correction performed. Shown are the resultant UMAPs, annotated for cell types (B) or for dataset of origin (C). Leptomeningeal clusters 0 and 4 are annotated based upon analyses included in this manuscript. The other clusters are comprised of transcriptomes from Pietilä et al. (2023), and are thus annotated as in that paper. **(D)** Violin plot showing the number of transcripts per cell (nCount) in each of the datasets included in the merged dataset in (B, C). Each dot represents an individual transcriptome. **(E)** UMAP as in (B) overlaid for expression of two mRNAs, *Aldh1a2,* and *Crabp2* known to be expressed in the developing leptomeninges. Expression levels are color-coded as per the adjacent keys. **(F)** UMAP cluster visualization of the leptomeningeal transcriptomes from the Xenium mesenchymal probeset dataset (Fig. 4C), shown at higher resolution and annotated for potential pial versus arachnoid cells. **(G)** Violin plots showing relative expression levels of two pial mRNAs, *Lamc3* and *Bmp2* and two arachnoid mRNAs, *Bnc2* and *Dpp4* in the pial versus arachnoid cells as annotated in (F). Red and green denote arachnoid and pial cells, respectively. Each dot corresponds to expression in an individual cell. **(H)** Spatial plots of the cortical interface and midline showing distribution of the pia and arachnoid cells annotated in (F). Note that pial cluster 9 cells (red) but not arachnoid cluster 6 or 11 cells (blue and black) extend down the midline. Download Figure 6-1, TIF file.

10.1523/ENEURO.0046-25.2025.f6-2 Figure 6-2 ***Genes differentially expressed in pial versus arachnoid leptomeningeal cells*.** Differential gene expression analysis was performed on the arachnoid and pial cell shown in Fig. 6A. Differentially expressed gene were defined as those expressed in >10% of cells in either group with a Bonferroni adjusted p-value < 0.05 (Adj. p-value) and ≥ 1.3 average log2 fold change (Avg log2FC). M = Mesenchymal Panel, B = Brain Panel. Download Figure 6-2, XLS file.

We compared the potential pial and arachnoid cells by differential gene expression analysis (Extended Data Fig. 6-2). This analysis identified 555 differentially expressed genes (average log2 fold change ≥1.3, adj *p* value <0.5, expression in at least 10% of cells), with 454 and 101 enriched in the potential pial versus arachnoid cells, respectively. Since a subset of these mRNAs were targeted in the Xenium probesets (Extended Data Figs. 4-3, 6-2), we asked about their spatial expression profiles in brain interface leptomeningeal cells. Notably, spatial plots showed that the putative pia-enriched mRNAs, including *Coch*, *Lamc3*, *Gjb2*, *Igfbp4*, and *Cyp1b1*, were expressed in leptomeningeal cells that extended down the midline (Fig. 6*E*). In contrast, mRNAs enriched in the putative arachnoid cells, such as *Bnc2*, *Cdh1*, *Dpp4*, and *Rspo2*, were excluded from the midline (Fig. 6*F*). The pial marker *Lama1* and arachnoid marker *Ppp1r1a* displayed similar differential spatial localization (Fig. 6*E*,*F*).

We asked whether these gene expression patterns corresponded to transcriptionally distinct leptomeningeal populations within the Xenium analysis, as seen in the scRNA-seq. Higher resolution UMAP analysis of the brain probeset data identified two groups of leptomeningeal cells (Fig. 6*G*). One of these (cluster 6) included potential pial cells that extended down the midline and were enriched for *Lama1*, *Cyp1b1*, *Gjb2*, and *Igfbp4* (Fig. 6*H*,*I*). The other population (clusters 4 and 8) included potential arachnoid cells that were excluded from the midline and were enriched for *Dpp4*, *Slc47a1*, *Cdh1*, and *Opn3* (Fig. 6*H–J*). Similar results were obtained with a higher resolution analysis of the mesenchymal probeset data (Extended Data Fig. 6-1*F–H*), identifying pial cells that extended down the midline that were enriched for *Lamc3*, *Bmp2*, *Lama1*, and *Cyp1b1*, and arachnoid cells that were excluded from the midline and were enriched for *Bnc2* and *Dpp4*. Thus, the Xenium and scRNA-seq analyses define two leptomeningeal cell populations that have locations and transcriptional signatures consistent with their identification as pia versus arachnoid cells.

### Brain interface and layer 1 cells comprise different cellular neighborhoods

**Figure 7.**
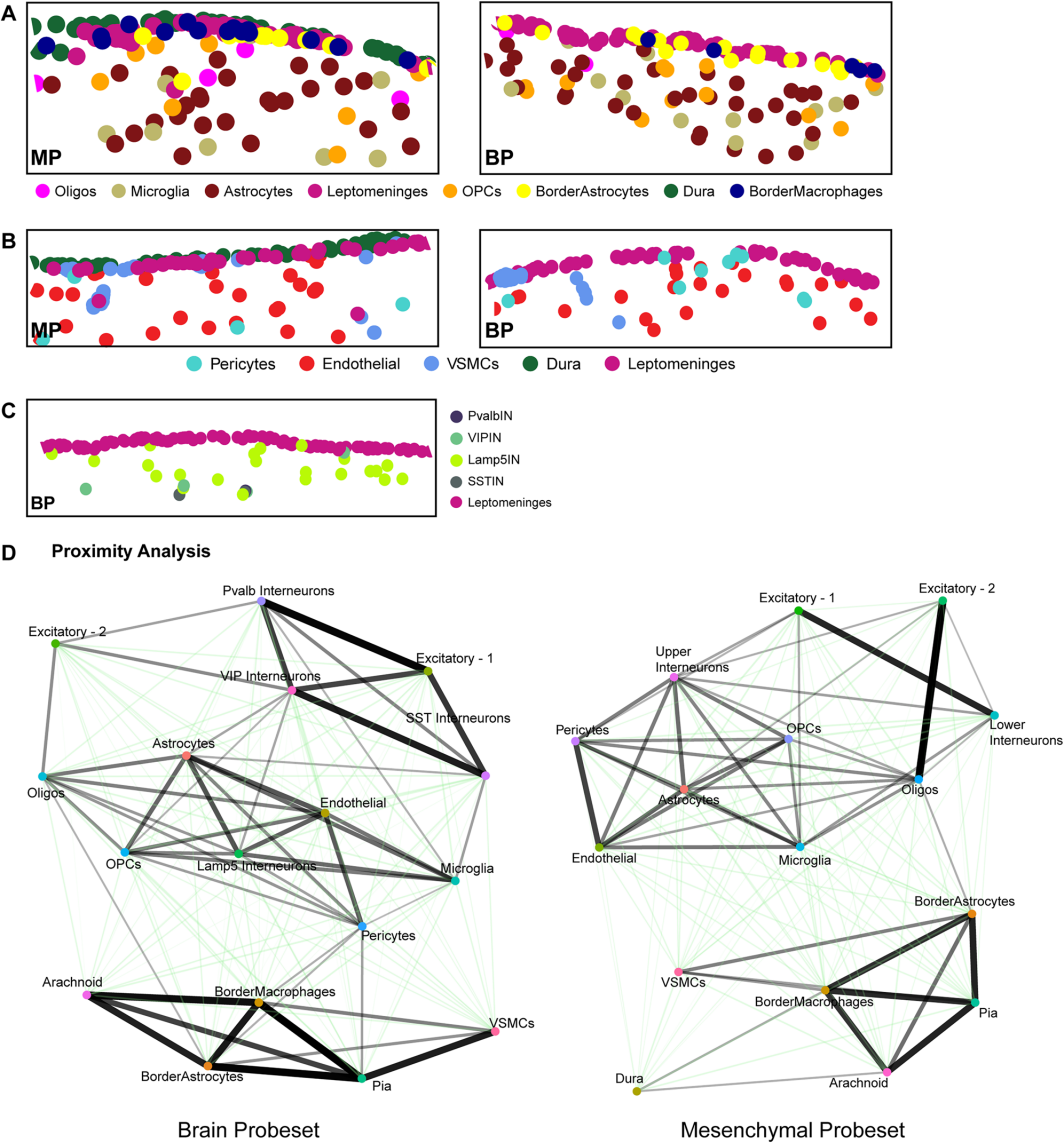
Single-cell spatial transcriptomic analysis defines distinct proximal cellular neighborhoods for the cortical brain interface and layer 1. Coronal adult cortical sections were analyzed by Xenium-based single-cell multiplexed in situ gene expression analysis with either a brain probeset targeting 347 genes or a mesenchymal probeset targeting 480 genes (Extended Data Fig. 4-3). The ROI and UMAPs of the resultant merged datasets are shown in Figure 4*A–C*. ***A***, Spatial plots of the cortical interface and layer 1 analyzed with the mesenchymal (MP, left) and brain (BP, right) probesets showing oligodendrocytes (pink), microglia (tan), parenchymal astrocytes (dark brown), leptomeninges (dark pink), OPCs (orange), border astrocytes (yellow), dural cells (green, mesenchymal probeset only), and border macrophages (dark blue). ***B***, Spatial plots of the cortical interface and layer 1 analyzed with the mesenchymal (MP, left) and brain (BP, right) probesets showing pericytes (turquoise), endothelial cells (red), VAMCs (blue), dural cells (green, mesenchymal probeset only), and leptomeninges (dark pink). ***C***, Spatial plot of the cortical interface and layer 1 analyzed with the brain probeset (BP) showing the leptomeninges (dark pink) and interneurons expressing *Lamp5* (lime green, Lamp5IN), *Vip* (blue-green, VIPIN), *Somatostatin* (gray, SSTIN), or *Parvalbumin* (black, PvalbIN). ***D***, Proximity analysis showing the relative strength of statistically significant interactions between different cell types located in the cortical brain interface and adjacent layer 1, as analyzed from the datasets obtained using either the brain probeset (left) or the mesenchymal probeset (right). The nodes are color coded and annotated for cell type, and the weight of the line indicates the strength of the interaction. Black lines indicate significant interactions based on a permutation test, comparing our data to a null distribution created from the random permutation of cell labels with fixed positions (see Materials and Methods for details). Green lines indicate interactions that were not statistically significant. Excitatory neuron-1 and excitatory neuron-2 denote transcriptionally distinct excitatory neuron clusters. Cell centroid information was used to perform this analysis, with a proximal interaction being defined as one where a cell centroid was within 70 µm of another cell centroid. This unsupervised analysis identifies two neighborhoods, the cortical brain interface and layer 1, as well as a partial neighborhood centered around the layer 2 excitatory neurons at the boundary of layer 1. Oligos, oligodendrocytes. Low magnification spatial plots (***A–C***) were generated using Seurat, and each dot represents the centroid of one cell. The proximity maps in ***D*** were generated using Giotto.

We next compared the cellular environments of the brain interface and the adjacent cortical layer 1. To do so, we used the mesenchymal and brain probeset spatial transcriptomic datasets to define the cortical layer 1 cellular environment. In both cases, spatial plots showed that layer 1 was relatively cell sparse and included scattered microglia, astrocytes, OPCs, and oligodendrocytes as well as vasculature-associated endothelial and mural cells (Fig. 7*A*,*B*). As indicated above, both probesets identified interneurons but not excitatory neurons in layer 1, as compared with layer 2, which was densely packed with both excitatory and inhibitory neurons (data not shown). The brain probeset identified a more abundant *Lamp5*-expressing interneuron population localized throughout layer 1 and a second less abundant *Vip*-expressing population largely located closer to the layer 2 border (Fig. 7*C*). As predicted, this analysis also identified interneurons in layer 2 but not layer 1 that expressed *Sst* or *Parvalbumin* (Fig. 7*C*; Lim et al., 2018; Yao et al., 2021).

We then more quantitatively defined cells in close proximity to each other within the brain interface/layer 1 environments using *cellProximityEnrichment* in Giotto v. 4.0.2 (described in detail in Dries et al., 2021) to analyze the single-cell spatial transcriptomic data. We defined cells as being proximal if their cell centroids were within 70 µm of each other and termed each proximal association an interaction. For both the mesenchymal and brain probeset data, this analysis identified two distinct cellular neighborhoods (Fig. 7*D*). One of these included the brain interface cells, the leptomeningeal pia and arachnoid cells, border astrocytes, and border macrophages, all of which were located in very close proximity to each other. Also in the vicinity were the VSMCs and, in the case of the mesenchymal probeset, the dura. In contrast, the brain interface cells were largely not significantly associated with other layer 1 cell types, although spatial plots did detect the occasional neural cell close to the interface (Fig. 7*A*).

The second proximal neighborhood included the cortical layer 1 and layer 2 cells other than border astrocytes: various interneurons, OPCs, oligodendrocytes, microglia, parenchymal astrocytes, excitatory neurons at the layer 1/2 border, as well as the endothelial cells and pericytes associated with smaller vessels and capillaries. With the exception of interactions involving layer 2 excitatory and inhibitory neurons, these interactions were largely not as robust as those between the border interface cells, consistent with the relatively sparse cellular density in layer 1 (Schuman et al., 2021). Thus, the cellular environments of the brain interface and cortical layer 1 are distinct, and the brain interface environment is likely predominantly determined by the border astrocytes, border macrophages, and leptomeningeal cells.

### Leptomeningeal cells express ligands predicted to act on both border astrocytes and macrophages

We next defined ligands made within the brain interface that might influence the biology of this cell-dense neighborhood. To do this, we used the scRNA-seq datasets (Fig. 1*A*,*B*) and a previously published ligand–receptor database (Toma et al., 2020). We defined a ligand mRNA as expressed if it was detectable in at least 5% of the relevant cell type. We identified 65 leptomeningeal ligands, 48 border macrophage ligands, and 54 border astrocyte ligands (Extended Data Fig. 8-2). Of these, 15 were expressed by all three cell types, including ligands such as *Bmp6*, *Efnb1*, *Hbegf*, *Il18*, *Vegfb*, and *Gas6*. Fifteen were expressed by leptomeninges and border astrocytes, including *Ctf1* and *Gdf11*, 12 were expressed by leptomeninges and border macrophages, including *Wnt5a*, *Igf2*, and *Tgfb1*, while one, *Sema4d*, was expressed by border astrocytes plus macrophages. The remaining ligands were expressed by only one of the three cell types; leptomeninges expressed 13 distinct ligands including *Bmp4*, *Bmp5*, *Bmp7*, *Sema3a*, and *Igf2*, border macrophages expressed 9 distinct ligands including *Osm*, *Tnf*, and *Il1b*, while border astrocytes specifically expressed 13 ligands, including *Fgf1*, *Tgfb2*, and *Wnt7a*, and the semaphorins *Sema3b*, *Sema4g*, and *Sema5b*.

**Figure 8.**
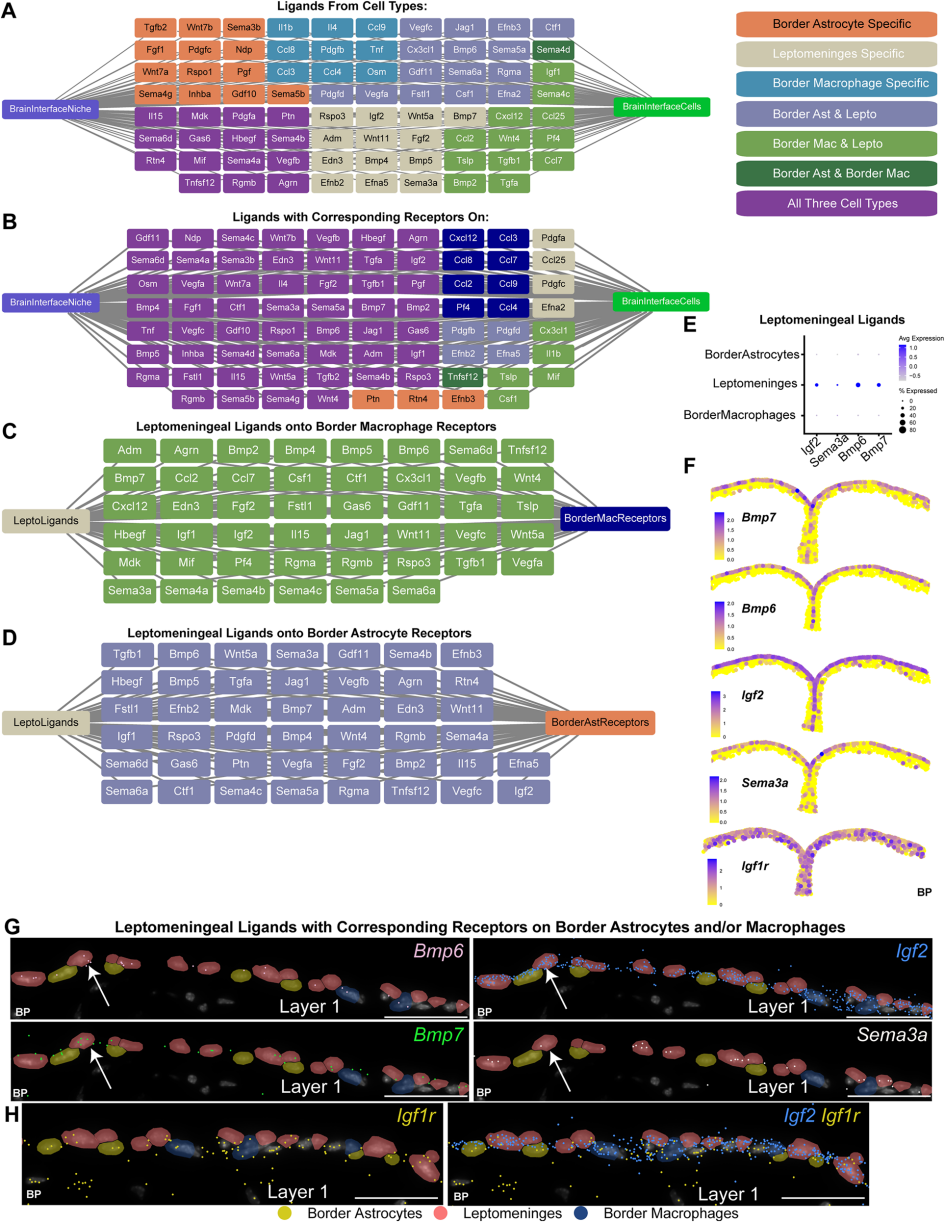
Leptomeningeal cells express ligands predicted to regulate border astrocytes and border macrophages. Also see Extended Data Figures 8-1 to 8-4. ***A–D***, Leptomeningeal cells, border astrocytes, and border macrophages were analyzed for their expression of ligand and ligand receptor mRNAs using the scRNA-seq data (Extended Data Figs. 8-2, 8-3, and 8-4). Ligand or receptor mRNAs were included if they were expressed in ≥5% of the relevant cell type. The data were used to generate predictive models of the bioactive ligand environment within the brain interface neighborhood. Each box includes a ligand known to bind to a corresponding receptor expressed by at least one of the brain interface cell types. ***A***, ***B***, All of the ligands made by the brain interface cells that have a receptor expressed by at least one of the interface cell types. In ***A***, these are color coded to show the cells that express these ligands, while in ***B***, they are color coded to show the cells that express the relevant receptor and thus are predicted to respond to the ligands. ***C***, ***D***, Models with ligands expressed by the leptomeningeal cells that have corresponding receptors on either border macrophages (***C***) or border astrocytes (***D***). ***E***, Dot plot showing expression levels of *Igf2*, *Sema3a*, *Bmp6*, and *Bmp7* mRNAs in leptomeningeal cells, border astrocytes, and border macrophages, as determined from the scRNA-seq data. The size of the dot indicates the percentage of cells detectably expressing the mRNA, and the color indicates relative expression level, coded as per the adjacent keys. ***F***, Spatial plots of *Bmp7*, *Bmp6*, *Igf2*, *Sema3a*, and *Igf1r* mRNAs in all brain interface and layer 1 cells analyzed used the brain probeset (BP), all shown on the same representative section. Relative mRNA expression levels are coded as per the adjacent keys. ***G***, High-resolution Xenium Explorer images of the cortical interface region analyzed with the brain probeset (BP) showing expression of *Bmp6* (light pink dots), *Igf2* (blue dots), *Bmp7* (green dots), *Igf2* (blue dots), and *Sema3a* (white dots) mRNAs relative to the leptomeningeal cells (pink), border astrocytes (yellow), and border macrophages (dark blue). The same field of view is shown in each panel, and arrows denote the same leptomeningeal cell detectably coexpressing multiple ligand mRNAs. Nuclei of other cell types (white) are also shown. ***H***, High-resolution Xenium Explorer images of the cortical interface region analyzed with the brain probeset (BP) showing expression of *Igf2* (blue dots) and *Igf1r* (yellow dots) mRNAs relative to the same cell types as in ***G***. Nuclei of other cell types (white) are also shown. Low magnification spatial plots in ***F*** were generated using Seurat and the high-resolution spatial plots in ***G*** and ***H*** with Xenium Explorer. Scale bars, 10 μm.

To ask if these ligands might influence the brain interface neighborhood, we used the same scRNA-seq data and ligand–receptor database to define receptors expressed by each interface cell type. This analysis defined 102, 95, and 79 receptors expressed by the leptomeninges, border astrocytes, and border macrophages, respectively (Extended Data Fig. 8-3). We used this information to predict which ligands might be biologically active. This modeling (Fig. 8*A*,*B*; Extended Data Fig. 8-4) predicted 78 ligand–receptor interactions within the brain interface neighborhood. Most ligands (52) had receptors on all three brain interface cell types, but the remainder were more specific.

10.1523/ENEURO.0046-25.2025.f8-1 Figure 8-1 ***Specific expression of leptomeningeal ligands, as analyzed by scRNA-seq.*** UMAPs showing the cortical surface-associated dataset from Fig. 1A, overlaid for expression of four ligands that are highly-enriched in leptomeningeal cells, *Sema3a, Bmp6, Bmp7* and *Igf2*. Expression levels are color-coded as per the adjacent keys. Download Figure 8-1, TIF file.

10.1523/ENEURO.0046-25.2025.f8-2 Figure 8-2 ***Analyses of leptomeningeal cell, border macrophage and border astrocyte ligand mRNA expression*.** Shown are ligand mRNAs expressed in leptomeningeal mesenchymal cells, border macrophages and border astrocytes at the cortical interface as determined by scRNA-seq, as well as the percentage of a given cell type that detectably expresses that ligand. For leptomeningeal cells and border macrophages ligands were extracted from the cortical surface-associated and bone-associated scRNA-seq datasets shown in Fig. 1A and B, and for border astrocytes from the merged astrocyte scRNA-seq dataset shown in Fig. 2A based on a curated ligand and associated receptor database (Toma et al., 2020). Ligands were only considered for further analysis (such as in the ligand-receptor modeling in Figure 8-4) if they were detectably expressed in at least 5% of the relevant cell type. Download Figure 8-2, XLS file.

10.1523/ENEURO.0046-25.2025.f8-3 Figure 8-3 ***Analyses of leptomeningeal cell, border macrophage and border astrocyte receptor mRNA expression*.** Shown are receptor mRNAs expressed in leptomeningeal mesenchymal cells, border macrophages and border astrocytes at the cortical interface as determined by scRNA-seq, as well as the percentage of a given cell type that detectably expresses that ligand. For leptomeningeal cells and border macrophages receptor mRNAs were extracted from the cortical surface-associated and bone-associated scRNA-seq datasets shown in Fig. 1A and B, and for border astrocytes from the merged astrocyte scRNA-seq dataset shown in Fig. 2A based on a curated ligand and associated receptor database (Toma et al., 2020). Receptors were only considered for further analysis (such as in the ligand-receptor modeling shown in Figure 8-4) if they were detectably expressed in at least 5% of the relevant cell type. Download Figure 8-3, XLS file.

10.1523/ENEURO.0046-25.2025.f8-4 Figure 8-4 ***Details of ligand-receptor communication models****.* Predicted ligand and receptor communication models were generated using the leptomeningeal, border macrophage and border astrocyte ligand and receptor mRNA lists shown in Figure 8-2 and 8-3. Shown are ligand-receptor communication models of (8-4i) ligands from all cells predicted to act on leptomeningeal, border macrophage or border astrocyte receptors, (8-4ii) leptomeningeal ligands predicted to act on border macrophage receptors, and (8-4iii) leptomeningeal ligands predicted to act on border astrocyte receptors. Download Figure 8-4, XLS file.

We also more specifically modeled ligands made by leptomeningeal cells that could influence their neighboring border astrocytes and macrophages (Fig. 8*C*,*D*; Extended Data Fig. 8-4). Most leptomeningeal ligands were predicted to act on both neighboring cell types. Analysis of the scRNA-seq data showed that some of these ligand mRNAs were highly enriched in the leptomeningeal cells relative to other neural and non-neural cell types, including *Igf2*, *Bmp6*, *Bmp7*, and *Sema3a* (Fig. 8*E*; Extended Data Fig. 8-1*A*). Since probes for these same ligands were present in the Xenium panels, we analyzed their spatial expression. Spatial plots showed that all four ligands were highly enriched and coexpressed in leptomeningeal cells at the brain interface (Fig. 8*F*,*G*). There was also scattered expression of these ligands in some layer 1 cells (Fig. 8*F*), consistent with the scRNA-seq. Furthermore, the Xenium panels included probes for *Igf1r*, one of the main receptors for Igf2. Spatial plots for *Igf1r* confirmed that it was broadly expressed within the brain interface and adjacent layer 1 and that *Igf1r*-positive border astrocytes and macrophages were adjacent to *Igf2*-expressing leptomeningeal cells (Fig. 8*F*,*H*). Consistent with these findings, all four of these leptomeningeal ligands have been shown to directly affect astrocytes and macrophages (Ishikawa et al., 1995; Choe et al., 2014; Aluganti Narasimhulu and Singla, 2020) and cultured meningeal cells secrete all of these ligands (Ishikawa et al., 1995; Ohe et al., 1996; Niclou et al., 2003; Choe et al., 2012; Segklia et al., 2012). Thus, leptomeningeal cells express ligands that are predicted to directly influence their macrophage and border astrocyte neighbors and perhaps play a role in regulating the brain interface.

## Discussion

The meninges have been studied for many years, but we are only now gaining a cellular/molecular picture of this key interface tissue, largely due to the advent of high-resolution imaging approaches and single-cell transcriptomics (reviewed in Betsholtz et al., 2024). Most studies using these newer approaches have focused on the meninges and their vascular and immune cell components (Engelhardt et al., 2017; DeSisto et al., 2020; Rustenhoven et al., 2021; Como et al., 2023; Pietilä et al., 2023). Here we have instead focused on the interface between the leptomeninges and the adjacent neural tissue. We show that in the adult cortex, this interface is composed of a layer of border astrocytes adjacent to leptomeningeal cells that are intermingled with resident macrophages and the occasional penetrating blood vessel. On the CNS side these interface cells are not closely associated with other neural cells, although they may be in proximity to axons of passage in cortical layer 1. This brain interface environment is predicted to be rich in growth factors, including ligands known to regulate interface cell types in other contexts. Thus, our data define and provide a molecular/cellular resource for the cortical interface, a unique brain compartment composed of neural and non-neural cells that together regulate CNS interactions with the periphery (Lynn et al., 2011; Choe et al., 2012; Suter et al., 2017; Chou et al., 2018; Jordão et al., 2019; DeSisto et al., 2020; Utz et al., 2020; Mapunda et al., 2022; Como et al., 2023).

Data presented here confirm recent findings (DeSisto et al., 2020; Pietilä et al., 2023; Smyth et al., 2024) that the leptomeninges and dura are composed of distinct types of mesenchymal cells. The dural cells that line the skull have much in common with connective tissue stromal cells and periosteal mesenchymal cells in other tissues. In contrast, the leptomeningeal cells are transcriptionally distinct from other mesenchymal cells and are differentially enriched for tight junction (*Tjp1*, *Cdh5*) and gap junction (*Gjb2*, *Gjb6*) mRNAs, reflective of their unique biological function (Castro Dias et al., 2019; Derk et al., 2023; Betsholtz et al., 2024). We find that in the adult cortex the leptomeningeal pial and arachnoid cells are only a few cell layers thick and that they are transcriptionally more similar to each other than they are to dural cells. We are, nonetheless, able to distinguish them transcriptionally by expression of mRNAs like *Dpp4* and *Lama1* and to confirm previous reports that pia but not arachnoid cells line the cortical hemispheric midline and are present on penetrating blood vessels (Daneman and Prat, 2015; DeSisto et al., 2020; Bonney et al., 2022; Jones et al., 2023; Pietilä et al., 2023).

Our data also define an intriguing population of astrocytes that interact with leptomeningeal cells at the cortical interface, in agreement with previous findings (Liu et al., 2013; Mason et al., 2021; Hasel et al., 2025). These border astrocytes, which comprise the glia limitans superficialis, are enriched for 381 genes relative to cortical parenchymal astrocytes, including *Myoc* and *Gfap*. This unique astrocyte:pial cell relationship is reminiscent of astrocyte:vasculature cell interactions since in both cases astrocytic end feet interact with an extracellular matrix layer deposited by adjacent non-neural cells (Daneman and Prat, 2015; Rua and McGavern, 2018; Kadry et al., 2020). However, while the vasculature-associated astrocytes are implicated in formation and maintenance of the blood–brain barrier, the function of brain interface border astrocytes is largely unknown (Batiuk et al., 2020; Mason et al., 2021). One likely function is to partner with pial cells and provide a cellular border that ensures cells do not move between neural and non-neural compartments, analogous to an epithelium. However, developmental studies suggest that border astrocytes may be more than just structural. During embryogenesis, the end feet of cortical radial glial precursors form a similar interface with the leptomeningeal cells, and this interplay is essential for appropriate cortical development (Halfter et al., 2002; Myshrall et al., 2012; Chou et al., 2018).

One somewhat surprising finding reported here is that border astrocytes are the only CNS cell type in close proximity to leptomeningeal cells. One potential explanation for this finding is that astrocytic end feet spatially exclude other cell types from the interface. However, there may also be more active exclusion mechanisms. For example, we show that the known repulsive ligand Sema3b is highly enriched in border versus parenchymal astrocytes, and this molecule could serve to repel axons and other neural cell types from the interface region (Nawabi et al., 2010).

The third major cell type within the brain interface neighborhood is an intriguing population of leptomeninges-resident macrophages. As previously reported (Jordão et al., 2019; Sankowski et al., 2024), we find that these are transcriptionally distinct from both the cortical dural macrophages (data not shown) and from microglia within the cortical parenchyma. The spatial transcriptomic data show that these resident macrophages are embedded within the leptomeningeal layers, in close proximity to both leptomeningeal cells and border astrocytes. Given their location, these macrophages are ideally situated to play a role in barrier function and to act as a first-line response to injury (Pedragosa et al., 2018; Joost et al., 2019; Jordao et al., 2019). Our ligand–receptor modeling suggests they may also directly regulate leptomeningeal cell and border astrocyte biology via secretion of ligands like TNFα and IL1β. Since these ligands are known to regulate the biology of mesenchymal cells in other tissues, then their relative importance for interface biology will be an important future avenue of investigation (Magliozzi et al., 2019; Mokbel et al., 2024).

Our spatial transcriptomic data also provide a cellular level view of layer 1 cortex and define microglia, OPCs, parenchymal astrocytes, and Lamp5-positive interneurons as the major cellular residents. On the surface side, layer 1 is bordered by brain interface astrocytes and on the other side by relatively dense layer 2 excitatory neurons, interneurons, and oligodendrocytes, consistent with previous morphological studies (Yao et al., 2021). Notably, we find that layer 1 cells are largely not in close proximity to each other. Since layer 1 includes many axons of passage, then it is possible that axonal interactions comprise the major homeostatic association for most layer 1 cells. Moreover, the relative distance between border astrocytes and other layer 1 cells suggests that secreted ligands may be the major way the brain interface communicates with the underlying cortical parenchyma (Suter et al., 2017; Gesuita and Karayannis, 2021).

Our findings indicate that the brain interface is a unique cellular neighborhood composed of three major cell types, leptomeningeal cells, border astrocytes, and tissue-resident macrophages. It is likely that communication between these closely associated cellular players involves both cell:cell contacts and secreted ligands. Our modeling and spatial transcriptomics provide growth factor candidates for this communication. As one example, we show that *Bmp6* and *Bmp7* expression are highly specific to adult leptomeningeal cells, and others have shown that these ligands are secreted by cultured leptomeningeal cells (Niclou et al., 2003) and can regulate both astrocyte and macrophage biology (Sabo et al., 2009; Choe et al., 2012; Du et al., 2019; Aluganti Narasimhulu and Singla, 2020). As a second example, we show that *Sema3a* is specifically expressed by adult leptomeningeal cells, and others have shown that meningeal cells synthesize Sema3a protein (Niclou et al., 2003) and that it is anti-inflammatory for macrophages (Rienks et al., 2017) and regulates glial precursor biology (Sugimoto et al., 2001). Thus, our predictive cell interaction and growth factor models, together with the detailed cellular–molecular definition of interface cells and their locations, will provide the basis for future studies asking how interactions between these three distinct brain interface cell types allow them to maintain the specialized brain:periphery interface, keep cells from transitioning in and out of the brain, and repair/regenerate after brain injury.

## Synthesis

Reviewing Editor: Eran Perlson, Tel Aviv University Decisions are customarily a result of the Reviewing Editor and the peer reviewers coming together and discussing their recommendations until a consensus is reached. When revisions are invited, a fact-based synthesis statement explaining their decision and outlining what is needed to prepare a revision will be listed below. The following reviewer(s) agreed to reveal their identity: Julie Siegenthaler.

Thank you both very much. bellow is the synthesis:

This manuscript employs single-cell and spatial transcriptomics to characterize local niches at the brain surface, identifying potential signaling pathways at the interface between the adult mouse cerebral leptomeninges and para-leptomeningeal brain regions (layer 1). Through spatial transcriptomics, the authors define cell types on both the peripheral and brain sides of this interface and perform interactome analysis to predict ligand-receptor pairs that may mediate signaling between leptomeningeal and border-located cell types (macrophages, astrocytes). Given that much of the existing research on neural signals has focused on the developing CNS, this dataset represents a valuable resource for studying adult leptomeningeal-brain crosstalk. This study is well-executed, presenting novel insights into the transcriptomic landscape of leptomeningeal-brain interactions. The data are highly valuable, but some methodological clarifications, figure adjustments, and validation experiments would strengthen the manuscript. Addressing these concerns will improve clarity, reproducibility, and impact.

Major Comments:

1. Figure 1:

a. Add a diagram illustrating the method of surface-associated cell isolation.

b. Resize UMAP plots for better readability and remove distracting black lines.

c. Include missing Y-axis labels in violin plots (Figure 1C, D, I).

d. Clarify whether mice were perfused before single-cell dissociation; if not, note that T cells, B cells, and neutrophils may be blood-borne contaminants. Consider removing potential blood cells from UMAP plots for clarity.

e. Reclassify 'vascular-associated mesenchymal cells' as 'vascular smooth muscle cells' since markers used (Pdgfrb, Myh11, Mylk, Acta2) are specific to vascular smooth muscle cells.

f. The term 'mesenchymal' should be reconsidered; suggest using 'fibroblast,' 'dura cell,' or 'leptomeningeal cell' instead.

g. Define the cell type 'lepto2' in 1E, 1F CSA dataset-does this represent arachnoid barrier or dural border cells? Clarify differences between these two clusters.

2. Figure 2:

a. Provide a brief justification for the focus on astrocytes in the text, as the transition from Figure 1 is abrupt.

b. Remove distracting lines in Figure 2A; instead, add a key to differentiate colors.

c. Ensure cluster numbers in Slc1a3 and Cxcl14 feature plots are visible.

d. Explain how the score in Figure 2C was calculated in the Methods section. Consider moving this data to Extended Data 3 if its relevance is limited.

3. Figure 3:

a. Indicate in D-H that unstained portions contain tissue.

b. Clarify whether panels D and E show different tissue samples or an artifact of separation. Include a low-magnification overview demonstrating that these images originate from the same piece of tissue.

c. Add a border astrocyte marker to better link this figure with preceding data.

4. Figures 4 and 5:

a. Specify the rostral-caudal location of sections and discuss reproducibility across sections.

b. Remove distracting black lines in Figures 4B and 4C.

5. Figure 6:

a. In pg. 22, first paragraph: Distinguish between Raldh2/Crabp2+ arachnoid cells extending down the midline and Dpp4/Cdh1+ arachnoid barrier cells that do not. Improve resolution to clarify upper vs. lower leptomeninges.

b. Consider integrating available meninges datasets to validate Lama1/Slc22a6 (pia) and Cdh1/Dpp4 (arachnoid).

c. Investigate whether the midline-extending pia is molecularly distinct from pia directly underlying the upper meninges.

6. Figure 7:

a. Provide validation of receptor expression to match ligand expression, e.g., show receptor localization for BMP ligands in Figure 7G.

7. Gene Signatures and Methodological Justification:

a. The rationale for selecting 50 genes for leptomeningeal and dural mesenchymal cell signatures is unclear-was an optimization analysis performed?

b. Justify the number of sections per mouse used in the study.

c. Ensure all cell markers listed in the Methods (p7, p11) are properly referenced.

Minor Comments:

• Add line numbers to text and figure number annotations for future submissions.

• Introductory paragraph on human meninges profiling should include PMID: 39657259 (recent publication on human fetal leptomeninges) and the preprint on human spatial meninges (https://doi.org/10.1101/2024.11.13.623440).

• Clarify terminology: 'peripheral side' of the leptomeninges may be misleading, as 'periphery' often refers to regions outside the CNS. Instead, specify 'non-parenchymal side' or define 'periphery' within the introduction.

• The acronym 'BP' is used inconsistently to describe both 'brain probeset' (Figure 6E-F) and 'Biological Process' (Extended Data tables). Use distinct acronyms to avoid confusion.

• Typographical errors: p.14 'FACs' should be 'FACS'.

## Author Response

Dear Dr. Perlson:

Please find attached our revised version of eNeuro eN-NWR-0046-25, "Single cell approaches define the murine leptomeninges as a distinct cellular neighborhood comprised of neural and nonneural cell types" by Ebert et al. We were delighted that you and the reviewers felt that this manuscript was potentially suitable for publication in eNeuro following appropriate revisions. To address the comments and concerns raised in the consensus review, we have substantively rewritten the manuscript and added considerable new data analysis to the manuscript. Of particular note, we have added a new section (including new Figures 6, 6-1 and Extended Table 6) distinguishing pial and arachnoid cells in the scRNA-seq and single cell spatial transcriptomic data. Please note that we have also submitted all of the new datasets described in this manuscript to the GEO public repository, and the accession numbers will be available in the very near future.

More specifically, to address the consensus reviewer comments, we have revised the manuscript as follows, with the reviewer comments in black and our responses in blue.

Major comments 1. Figure 1: a. Add a diagram illustrating the method of surface-associated cell isolation.

As requested, we have now added a schematic to Fig. 1-1A. b. Resize UMAP plots for better readability and remove distracting black lines.

We have now resized the UMAP plots in this figure. We have also removed the black lines and have replaced them with light grey hatching. This approach allows us to also show the color-coded clusters in the same UMAP plot. c. Include missing Y-axis labels in violin plots (Figure 1C, D, I).

We have now included these. d. Clarify whether mice were perfused before single-cell dissociation; if not, note that T cells, B cells, and neutrophils may be blood-borne contaminants. Consider removing potential blood cells from UMAP plots for clarity.

The mice we used for the scRNA-seq analyses were not perfused, and thus the immune cells may indeed come from the blood. We have clarified this in the results text (line 452-453). e. Reclassify 'vascular-associated mesenchymal cells' as 'vascular smooth muscle cells' since markers used (Pdgfrb, Myh11, Mylk, Acta2) are specific to vascular smooth muscle cells.

As requested, we have now reclassified the vascular smooth muscle cells throughout the text. As one example, see line 451. f. The term 'mesenchymal' should be reconsidered; suggest using 'fibroblast,' 'dura cell,' or 'leptomeningeal cell' instead.

We thank the reviewers for asking us to consider this issue. It is true that in the field meningeal cells are frequently called fibroblasts. However, it has also become increasingly clear that Pdgfra-positive mesenchymal cells are heterogeneous and include, for example, fibroblasts, different types of precursor cells and cells other than fibroblasts such as those in the bone lineage (for example, see Carr et al., 2019; Storer et al., 2020; Li et al., 2024). For consistency and specificity, in the manuscript we have now used the terms dural cells and leptomeningeal cells when their identity and location is known, and the term Pdgfra-positive mesenchymal cells when discussing these cells generally and/or when their identity is not known. We have clarified this in the results text (lines 467-471). g. Define the cell type 'lepto2' in 1E, 1F CSA dataset-does this represent arachnoid barrier or dural border cells? Clarify differences between these two clusters.

In response to this question, we reanalyzed this scRNA-seq subset, and found that the differences between lepto1 and lepto2 were modest and were not consistent with arachnoid barrier or dural border cells. Moreover, if we reduced the resolution on the UMAP plot only slightly, these two clusters resolved into a single cluster. We have now included this slightly lower resolution in the manuscript, as shown in the UMAP plot in Fig. 1E and in the related Tables 1-1 and 1-2.

Having said that, as a further response to this question and to several of the other reviewer comments, we increased the power of our analyses by merging our dataset with previously published brain surface meningeal scRNA-seq data from Pietilä et al. (2023). This allowed us to define arachnoid versus pial leptomeningeal cells, as described in detail in the response to Figure 6, comment c below.

2. Figure 2: a. Provide a brief justification for the focus on astrocytes in the text, as the transition from Figure 1 is abrupt.

We have now done this (see lines 510-513). b. Remove distracting lines in Figure 2A; instead, add a key to differentiate colors.

We have now removed the black lines and have replaced them with light grey hatching as an alternative to using a color key. This approach allowed us to show the color-coded clusters in the same UMAP plot. c. Ensure cluster numbers in Slc1a3 and Cxcl14 feature plots are visible.

We have now done so. d. Explain how the score in Figure 2C was calculated in the Methods section. Consider moving this data to Extended Data 3 if its relevance is limited.

We have now explained how we generated the transcriptional signatures in the Materials and Methods (lines 203-210).

3. Figure 3: a. Indicate in D-H that unstained portions contain tissue. b. Clarify whether panels D and E show different tissue samples or an artifact of separation.

Include a low-magnification overview demonstrating that these images originate from the same piece of tissue.

To address these two comments, we have now added low magnification images of the relevant tissue sections to Fig. 1E and F (see the figure and lines 1355-1358 and 1363-1367) to show that the space between the dura and leptomeninges is indeed artefactual in these particular brain sections. c. Add a border astrocyte marker to better link this figure with preceding data.

The best border astrocyte immunostaining marker that we have identified is GFAP, and we have therefore added a high magnification image of this in Fig. 3C (see lines 1348-1350). We have relied upon the combined scRNA-seq and Xenium analyses to confirm other marker genes expressed by the border astrocytes.

4. Figures 4 and 5: a. Specify the rostral-caudal location of sections and discuss reproducibility across sections.

We have made two additions to address this comment. First, in Fig. 4-1A and B we now show images of coronal cortical sections at the levels used for the Xenium analyses (also see lines 612-620 and 1603-1614). All of the mesenchymal probeset sections and the brain probeset sections analyzed here were at a very similar rostro-caudal level as shown in Fig. 4-1A. The previously published Xenium datasets that we analyzed here (GEO GSE266689, sections GSM8647390, GSM8647391 and GSM8647392) were from three slightly more caudal sections (see Fig. 4-1B). Second, with regard to reproducibility, we identified the same cell types and spatial organization of the cortical interface for all sections/animals, regardless of any small differences in rostro-caudal level. To illustrate this we show (a) that transcriptomes from all sections analyzed with a given probeset merged very well without batch correction (see Fig. 4-1C and 4-2A), and (b) that the spatial organization of the different brain interface cell types is similar in multiple sections and at both rostro-caudal levels (compare new Fig. 4-2C to Fig. 4D and F; also see lines 634-635 and 1623-1629). b. Remove distracting black lines in Figures 4B and 4C.

We have removed the black lines and have replaced them with light grey hatching. This approach allows us to show the color-coded clusters in the same UMAP plot.

5. Figure 6: a. In pg. 22, first paragraph: Distinguish between Raldh2/Crabp2+ arachnoid cells extending down the midline and Dpp4/Cdh1+ arachnoid barrier cells that do not. Improve resolution to clarify upper vs. lower leptomeninges. b. Consider integrating available meninges datasets to validate Lama1/Slc22a6 (pia) and Cdh1/Dpp4 (arachnoid). c. Investigate whether the midline-extending pia is molecularly distinct from pia directly underlying the upper meninges.

We have addressed these three related comments by including a new section in the manuscript where we define arachnoid versus pial leptomeningeal cells using scRNA-seq and spatial transcriptomics. This new section (lines 232-246 and 678-716) includes two new figures (Figures 6 and 6-1) and a new table (Figure 6-3). More specifically, as suggested by the reviewers, we have taken advantage of previously published brain surface meningeal scRNA-seq datasets (Pietilä et al., 2023; GEO GSE227713) to augment the scRNA-seq datasets we obtained here. We chose two of the Pietilä et al., 2023 datasets that most closely matched our digest conditions, analyzed them using our pipeline, identified Pdgfra-positive mesenchymal cell transcriptomes, and then subsetted and reanalyzed these on their own. This analysis replicated the published analysis (seen in the far left panel of Fig. 1D in Pietilä et al., 2023), and identified several transcriptionally distinct meningeal mesenchymal cell populations (shown here in new Fig. 6-1A). We then merged these transcriptomes with our own dural and leptomeningeal cell transcriptomes (from Fig. 1E) and used two iterations of Harmony to correct for differences in read-depth (shown in new Fig. 6-1B-D). This analysis identified two leptomeningeal cell clusters as identified by their expression of leptomeningeal marker genes, including Crabp2 and Aldh1a1 (shown in new Fig. 6A-C and Fig. 6-1E). Both clusters included intermingled cells from the cortical brain surface and Pietilä et al. 2023 datasets (new Fig. 6A, B). One of these clusters was enriched for Lama1 and one for Ppp1r1a (new Fig. 6D), identifying them as potential pial versus arachnoid cells respectively.

We then compared these two clusters by differential gene expression (new Figure 6-2). Fortunately, a subset of mRNAs that were highly enriched in the pial versus arachnoid cell clusters were also targeted in our Xenium probesets. We took advantage of these differentially-expressed genes to reanalyze the Xenium datasets and in so doing identified arachnoid and pial cells in both the brain and mesenchymal probeset datasets (see Fig. 6E-J and Fig. 6-1F-H). Notably, with both probesets the pial cells were enriched for the pial mRNAs identified in the scRNA-seq, and spatial analyses showed that these cells extended across the brain surface and down the midline. Conversely, the arachnoid cells were enriched for the arachnoid mRNAs identified in the scRNA-seq and spatial analyses showed that while they were present on the brain surface they did not extend down the midline.

6. Figure 7: a. Provide validation of receptor expression to match ligand expression, e.g., show receptor localization for BMP ligands in Figure 7G.

Unfortunately, the BMP receptor mRNAs are not targeted by our Xenium probesets. However, our probesets did target Igf1r, which encodes one of the receptors for IGF2, and we have therefore now included spatial expression data for this receptor comparing it to the expression of Igf2 at the cortical brain interface (see new Fig. 8G and H, described in lines 785-788).

7. Gene Signatures and Methodological Justification: a. The rationale for selecting 50 genes for leptomeningeal and dural mesenchymal cell signatures is unclear-was an optimization analysis performed? The method used to generate the transcriptional signatures is now described in the Materials and Methods (lines 202-210). b. Justify the number of sections per mouse used in the study.

We included sections from 4 adult C57Bl/6 mice for analyses with the mesenchymal probeset and from 5 adult C57Bl/6 mice for analyses with the brain probeset. For the latter, two mice were analyzed in the current study and three were previously published in Willis et al. 2025 (GEO GSE266689, sections GSM8647390, GSM8647391, GSM8647392). However, the ROI examined here was not previously analyzed in Willis et al. 2025. For both probesets, one to two sections per mouse were analyzed by Xenium. Thus, we analyzed 4-5 independent biological replicates per probeset, consistent with current standards in the field (for example, see Willis et al., 2025; Ma et al., 2024). This is now explained in lines 263-274 of the Materials and Methods. c. Ensure all cell markers listed in the Methods (p7, p11) are properly referenced.

We have now added references for these cell markers (see lines 183-200 and lines 343-367).

Minor Comments: • Add line numbers to text and figure number annotations for future submissions.

We have now done this. • Introductory paragraph on human meninges profiling should include PMID: 39657259 (recent publication on human fetal leptomeninges) and the preprint on human spatial meninges (https://doi.org/10.1101/2024.11.13.623440).

We have added these two references as requested (see line 45). • Clarify terminology: 'peripheral side' of the leptomeninges may be misleading, as 'periphery' often refers to regions outside the CNS. Instead, specify 'non-parenchymal side' or define 'periphery' within the introduction.

We have changed this, and used the phrase 'non-parenchymal side' as requested (see line 52). • The acronym 'BP' is used inconsistently to describe both 'brain probeset' (Figure 6E-F) and 'Biological Process' (Extended Data tables). Use distinct acronyms to avoid confusion.

In the Extended Tables we have now used BioP as an acronym rather than BP. • Typographical errors: p.14 'FACs' should be 'FACS'.

We have corrected this.

Tables have now been updated in naming to follow eNeuro guidelines more closely. Extended Table 1 and 2 are now Figure 1-2 and 1-3, Extended Tables 3 and 4 are now Figure 2-2 and 2-3, and extended tables 7-9 are now Figure 8-2, 8-3, and 8-4. Extended Table 10 is now Figure 4-3.

We would like to thank you and the reviewers for your thoughtful comments since we feel that we have substantively improved the manuscript by addressing them. We hope that the manuscript is now acceptable for publication in eNeuro.

With kind regards, Freda Miller
