# Supplementary figures and images for: Single-Cell Approaches Define the Murine Leptomeninges: Cortical Brain Interface as a Distinct Cellular Neighborhood Composed of Neural and Non-neural Cell Types
Source: eNeuro. 2025 Aug 22;12(8):ENEURO.0046-25.2025. doi: 10.1523/ENEURO.0046-25.2025 (PMC12376965; doi:10.1523/ENEURO.0046-25.2025)

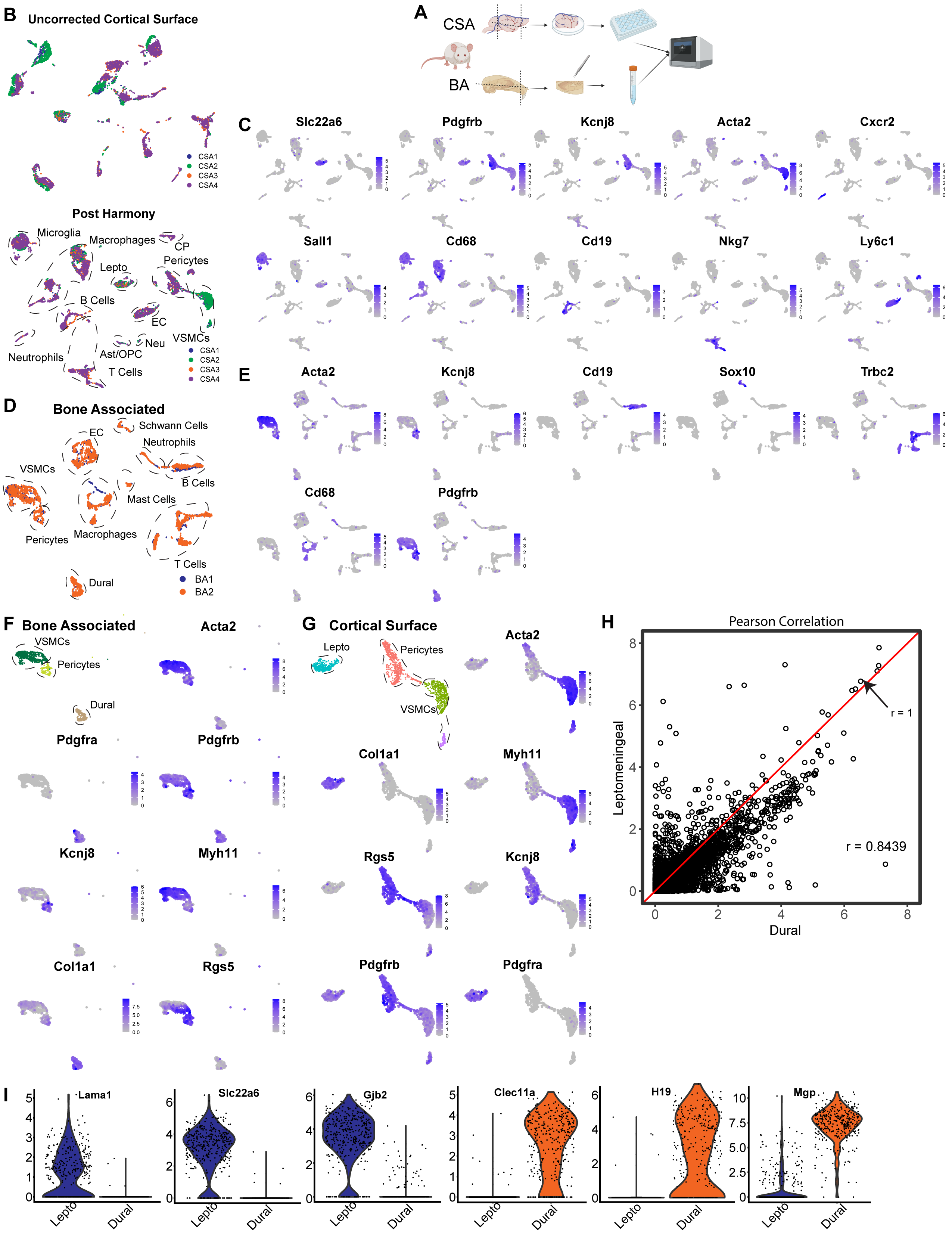

Supplement: Figure 1-1 — scRNA-seq to analyze adult murine cortical leptomeninges and dura-associated cell types. (A) Schematic of methods used to generate cells for the scRNA-seq datasets shown in Fig. 1A and B. The top illustrates the brain surface dissection and digestion and the bottom the bone-associated dissection. (B) UMAP visualization of merged transcriptomes from 4 independent cortical surface-associated (CSA) scRNA-seq runs (CSA1-4). Transcriptomes from each run are colored as per the adjacent legend. The top panel shows the cluster UMAP without Harmony batch correction, and the bottom with one iteration of Harmony. The bottom UMAP is also annotated for cell types. CP = choroid plexus, EC = endothelial cells, Lepto = leptomeninges, Ast/OPC = astrocytes and oligodendrocyte precursor cells, Neu = neurons, VSMC = vascular smooth muscle cells. (C) The batch-corrected, merged dataset in (B, bottom panel) was overlaid for expression of marker genes specific to different cell types. Expression levels are color-coded as per the adjacent keys. (D) UMAP visualization of merged transcriptomes from 2 independent bone-associated scRNA-seq runs (BA1, BA2). Transcriptomes from each run are colored as per the adjacent legend. The UMAP is also annotated for cell types. EC = endothelial cells. (E) The merged bone-associated dataset in (D) was overlaid for expression of marker genes specific to different cell types. Expression levels are color-coded as per the adjacent keys. (F, G) Pdgfrb-positive mesenchymal cells were subsetted from the bone-associated and cortical surface-associated datasets shown in (B) and (D), and reanalyzed. The annotated UMAPs (upper left panels) show the subsetted transcriptomes colored by cluster, and annotated for Pdgfra-positive dural or leptomeningeal cells (Dural in F, Lepto in G), and for Pdgfra-negative pericytes and vascular smooth mucle cells (VSMCs). The remainder of the panels show expression overlays for genes characteristic of each of the different mesenchymal [file eneuro-12-ENEURO.0046-25.2025-s002.tif]

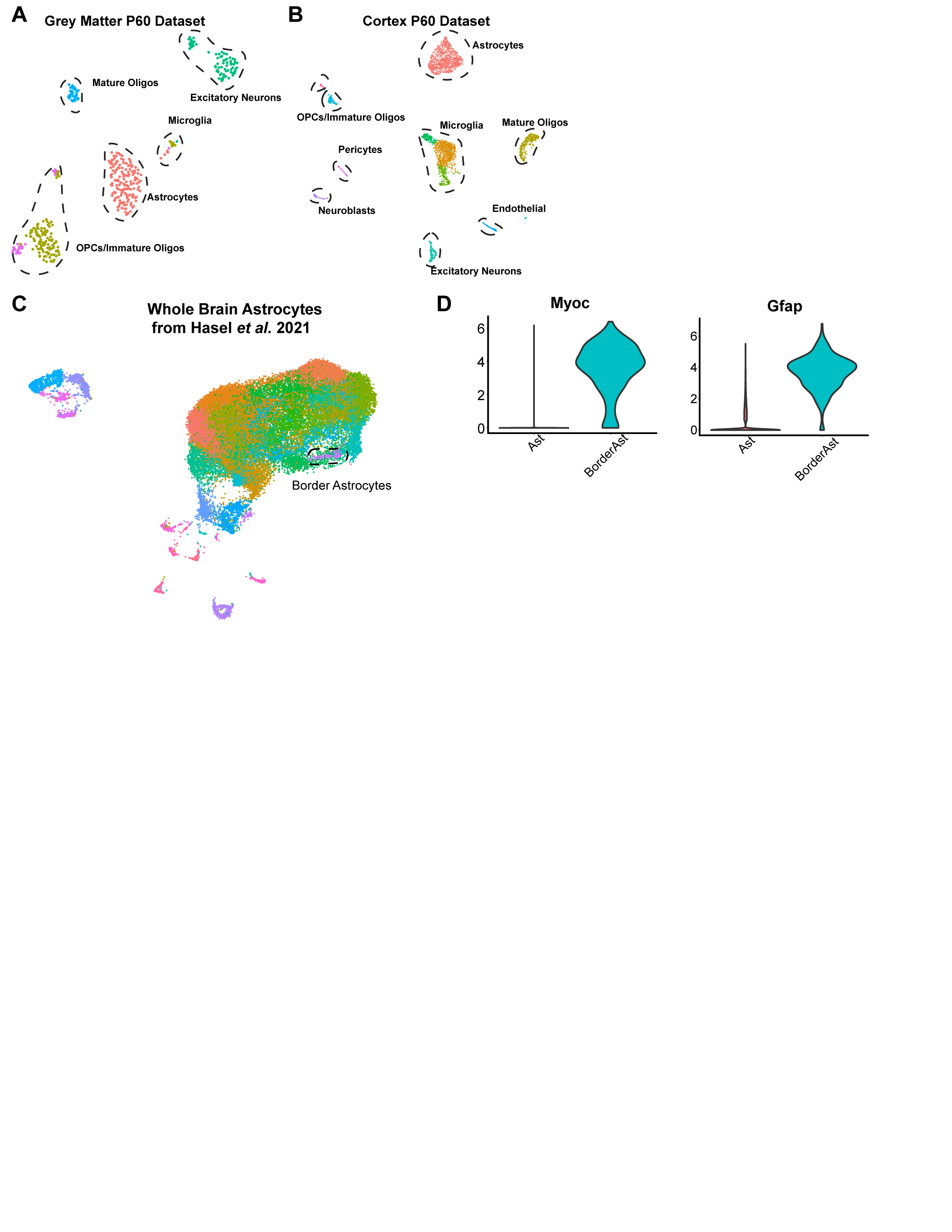

Supplement: Figure 2-1 — scRNA-seq to analyze adult cortical and border astrocytes. (A, B) UMAP visualizations of transcriptomes from previously-published (Dennis et al., 2024; GEO GSE255405) scRNA-seq datasets of dissected postnatal day 60 (P60) cortical grey matter (A) or total cortex (B) tissue. Transcriptomes were reanalyzed and cell types were identified using well-characterized marker genes. The transcriptionally-distinct clusters are color-coded, and different cell types annotated and denoted by the hatched lines. (C) UMAP visualization of transcriptomes from a previously-published (Hasel et al., 2021; GEO GSE148611) whole brain astrocyte scRNA-seq dataset. Transcriptomes were reanalyzed and transcriptionally-distinct clusters are color-coded. The cluster most enriched for Myoc and Gfap expression (outlined) was subsetted and used for subsequent analyses. (D) Violin plots showing relative expression levels of Myoc and Gfap mRNAs in the putative whole brain border astrocytes (BorderAst) from the cluster outlined in (C), versus all other astrocytes (Ast) from the dataset shown in (C). Download Figure 2-1, TIF file. [file eneuro-12-ENEURO.0046-25.2025-s005.tif]

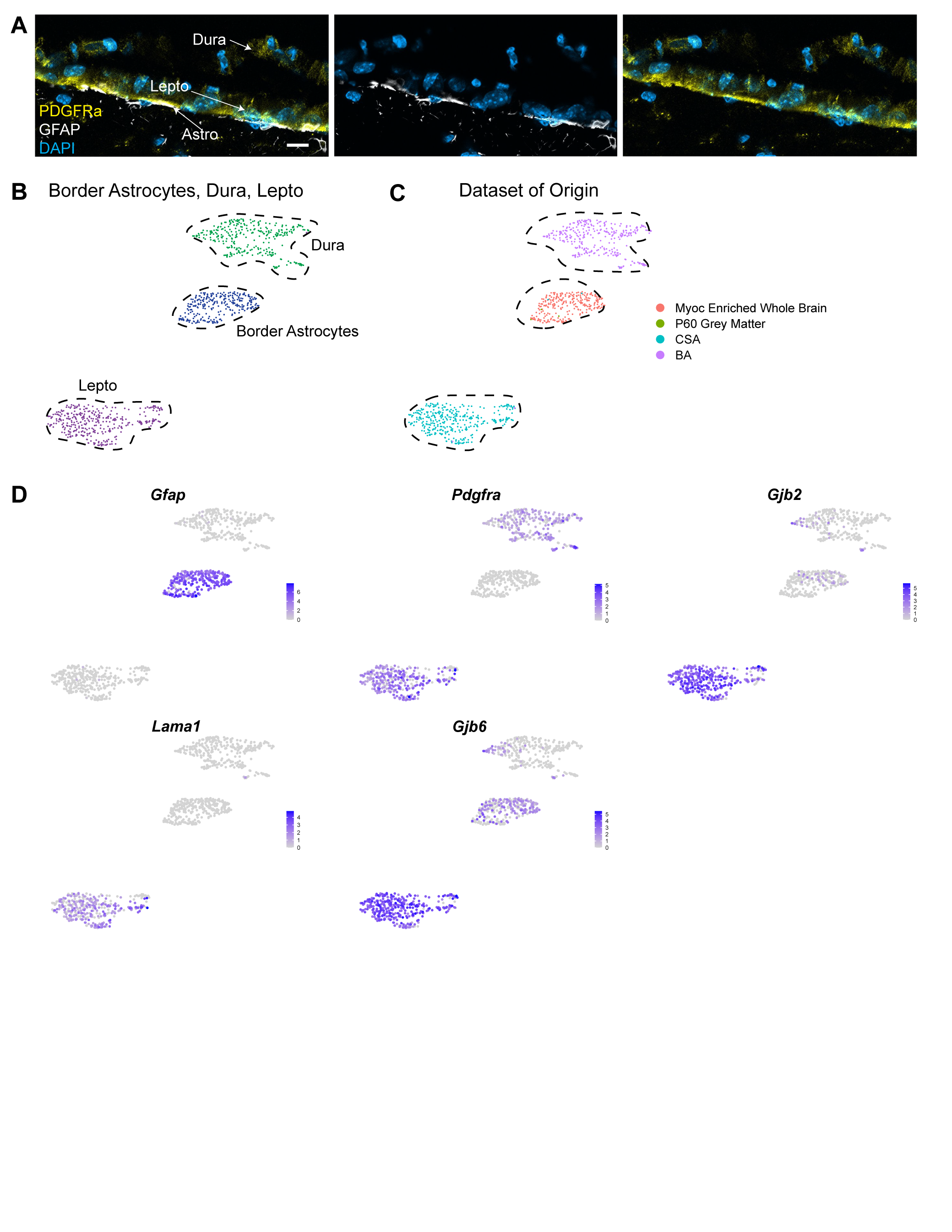

Supplement: Figure 3-1 — Characterization of the cortical brain interface by immunostaining. (A) High magnification confocal images of a sagittal section through the brain interface and skull, immunostained for PDGFRα (yellow) and GFAP (white), and counterstained with DAPI (blue). The left panel shows the merge and the right two immunostaining for PDGFRα or GFAP separately. Lepto = leptomeninges, Astro = border astrocytes. Scale bar = 10 μm. (B, C) The border astrocyte transcriptomes from cluster 2 in Fig. 2A and the leptomeningeal and dural cell transcriptomes from Fig. 1E were merged and reanalyzed. Shown are UMAPs with the clusters color-coded and annotated (B) or the datasets of origin indicated as per the adjacent color legend (C). CSA = cortical surface-associated dataset, BA = bone-associated dataset. (D) Gene expression overlays of the merged astrocyte plus meningeal mesenchymal cell dataset shown in (B, C) for selected genes that distinguish the different cell types. Expression levels are color-coded as per the adjacent keys. Download Figure 3-1, TIF file. [file eneuro-12-ENEURO.0046-25.2025-s008.tif]

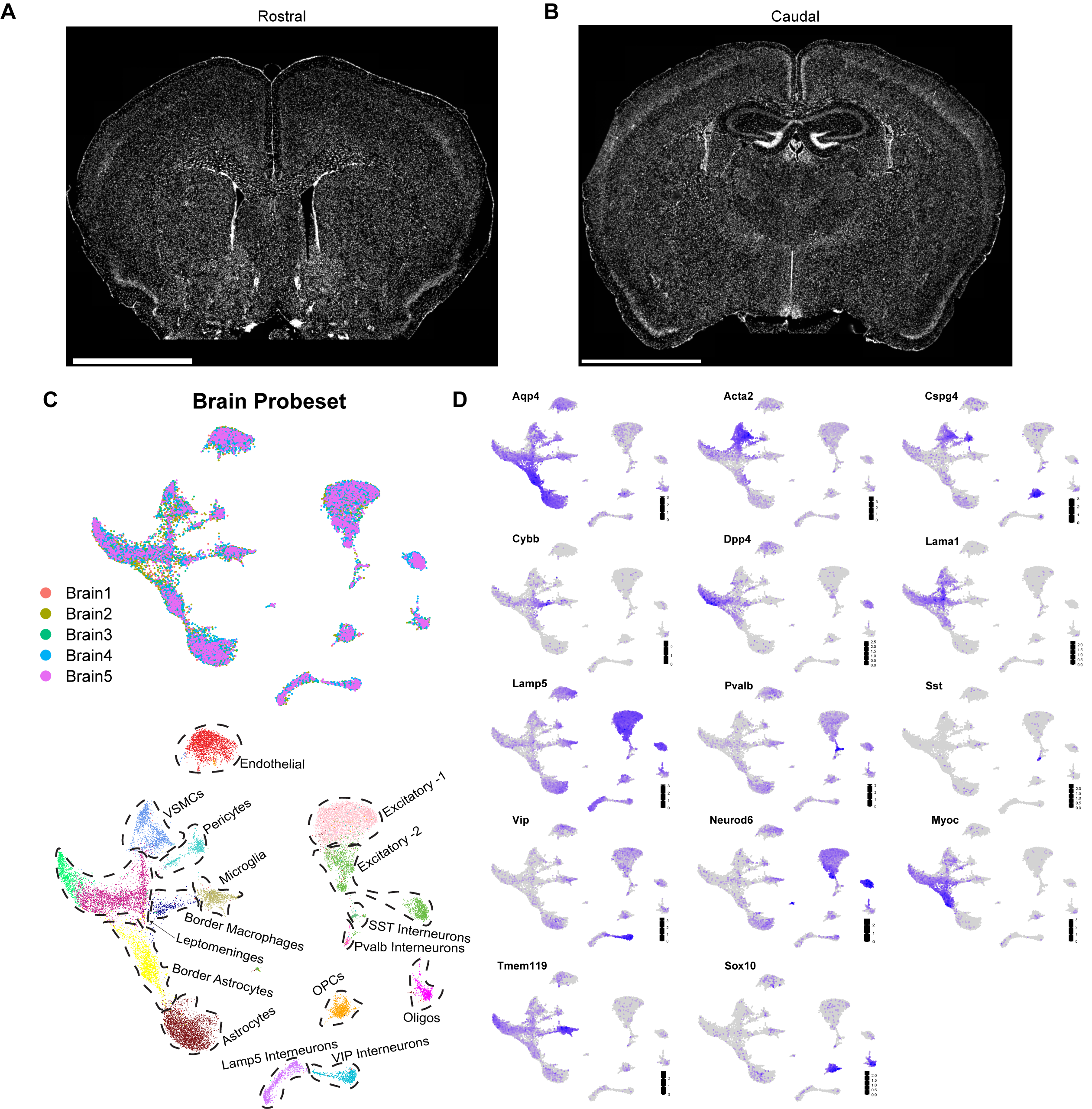

Supplement: Figure 4-1 — Analysis of the cortical interface and layer one cells using single cell multiplexed in situ gene expression analysis with the brain probeset. (A, B) Coronal cortical sections were analyzed by Xenium-based single cell multiplexed in situ gene expression analysis. (A) shows a representative Xenium Explorer image of a rostral brain section at the level used for all mesenchymal probeset analyses and for 2 of the 5 brains analyzed with the brain probeset. (B) shows a representative Xenium Explorer image of the more caudal brain sections used to generate the brain probeset data previously published in Willis et al. (2025; GEO GSE266689, sections GSM8647390, GSM8647391, GSM8647392). The ROI that was analyzed for all sections is shown in Fig. 4A, and the annotated UMAP cluster visualizations of the resultant merged transcriptomes are shown in Fig. 4B and C. (C) UMAPs of the merged brain probeset data showing either the annotated clusters (bottom; the same UMAP as in Fig. 4B) or the section of origin (top) for each of the transcriptomes. Brain1-5 denotes 5 sections from 5 different mice. Each dot represents a single transcriptome. VSMCs = vascular smooth muscle cells, Oligos = oligodendrocytes, SST = somatostatin, Pvalb = parvalbumin, VIP = vasoactive intestinal peptide. (D) Expression overlays for selected marker genes on the UMAPs shown in (C). Expression levels are color-coded as per the adjacent keys. Space bars = 2500 μM. Download Figure 4-1, TIF file. [file eneuro-12-ENEURO.0046-25.2025-s009.tif]

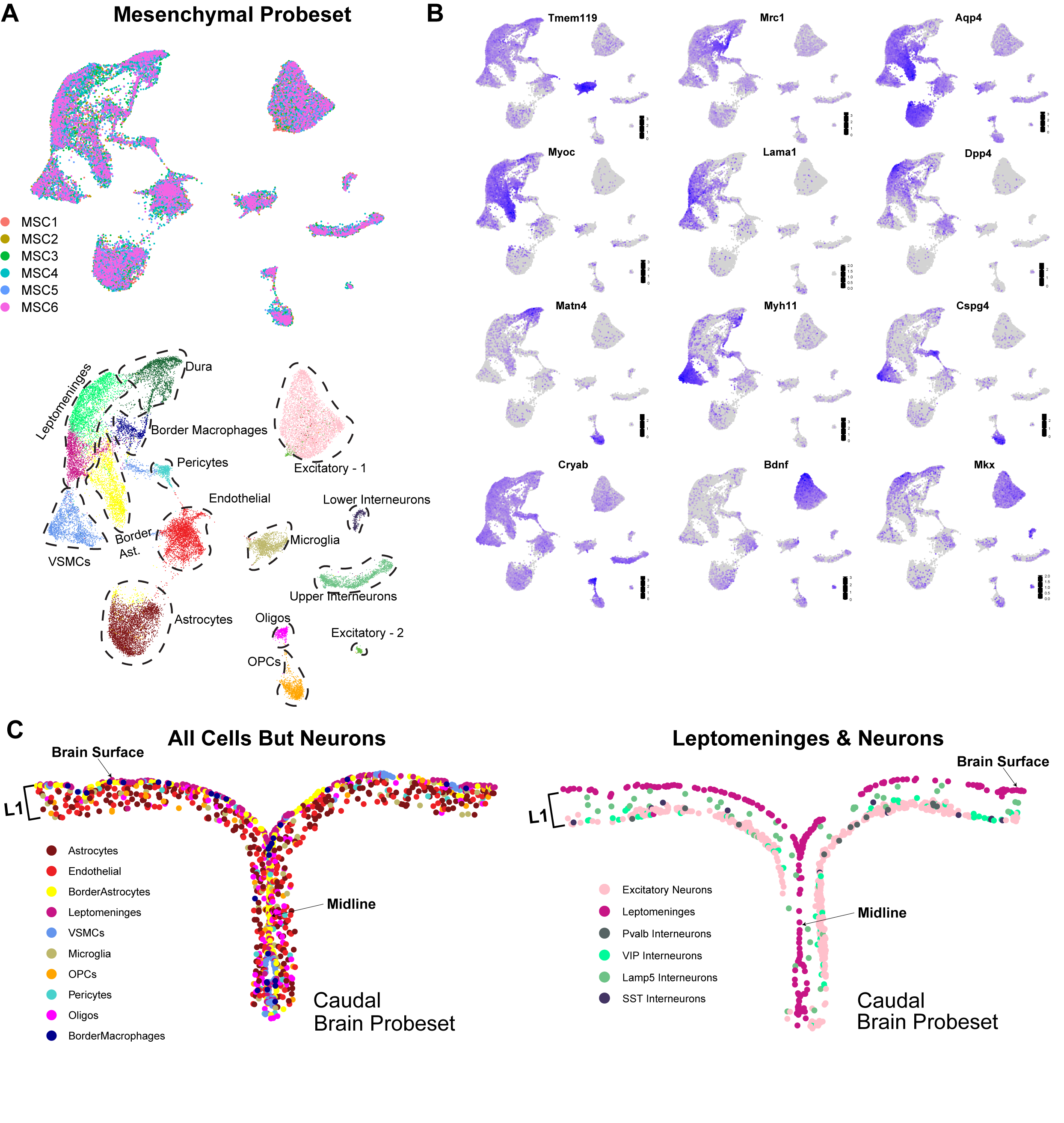

Supplement: Figure 4-2 — Analysis of the cortical interface and layer one cells using single cell multiplexed in situ gene expression analysis. (A, B) Coronal cortical sections as shown in Fig. 4-1A were analyzed by Xenium-based single cell multiplexed in situ gene expression analysis with the mesenchymal probeset. The ROI that was analyzed is shown in Fig. 4A. (A) UMAPs of the merged mesenchymal probeset data showing either the annotated clusters (bottom; the same UMAP as in Fig. 4C) or the section of origin (top) for each of the transcriptomes. MSC1-6 denotes 6 sections from 5 difference mice. Each dot represents a single transcriptome. VSMCs = vascular smooth muscle cells, Oligos = oligodendrocytes. (B) Expression overlays for selected marker genes on the UMAPs shown in (A). Expression levels are color-coded as per the adjacent keys. (C) Coronal adult mouse cortex sections at the level shown in Fig. 4-1B were analyzed by Xenium-based single cell multiplexed in situ gene expression analysis with the brain probeset. The ROI that was analyzed is shown in Fig. 4A. Shown are spatial plots of the midline and adjacent cortical interface and layer one from the ROI of a representative section illustrating all cell types except neurons (left) or leptomeninges and neurons (right), color-coded as per the legends. L1 denotes cortical layer one. Download Figure 4-2, TIF file. [file eneuro-12-ENEURO.0046-25.2025-s010.tif]

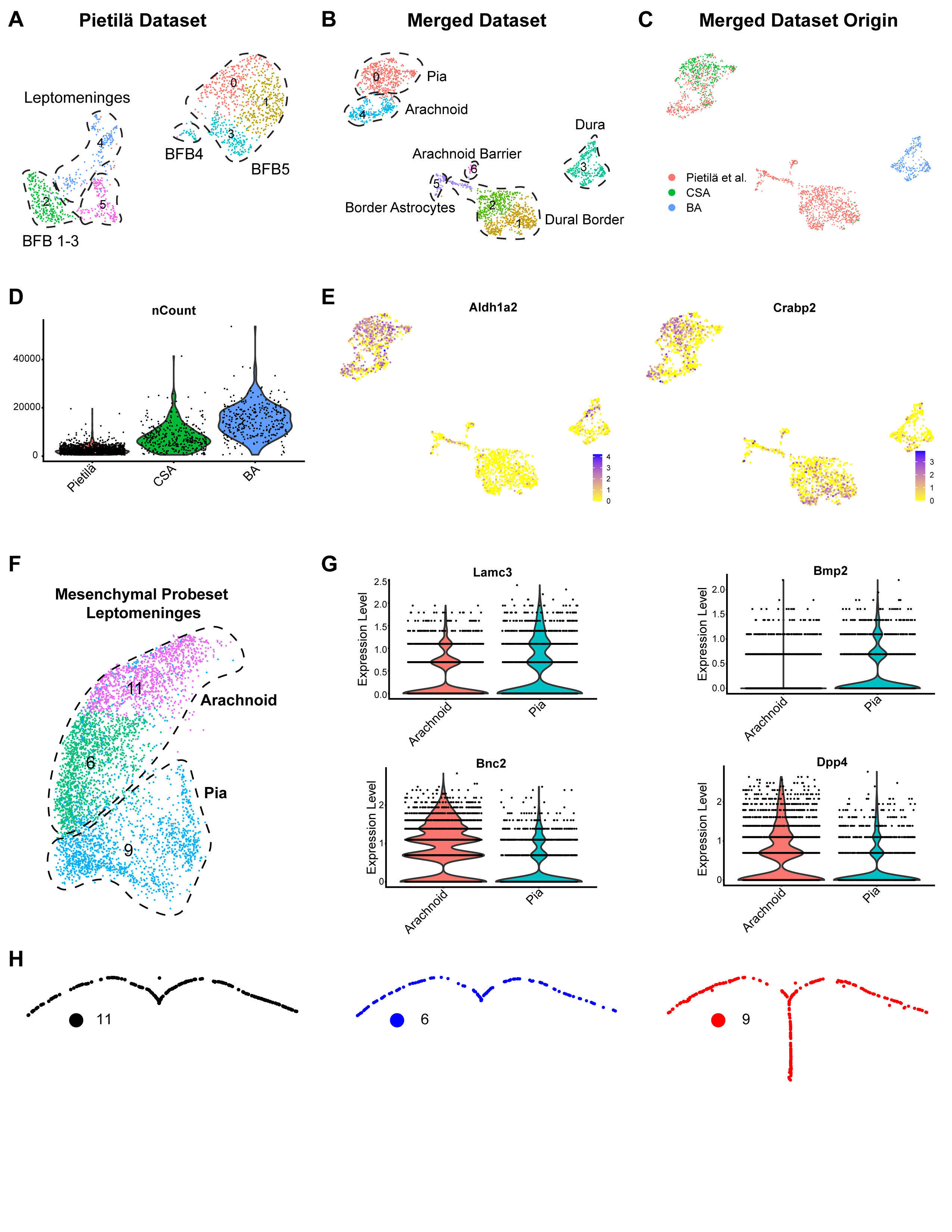

Supplement: Figure 6-1 — Spatially defining arachnoid and pial cells at the cortical brain interface. (A) Two adult mouse brain surface scRNA-seq datasets from Pietilä et al. (2023; GEO GSE227713) were run through our pipeline, and analyzed and annotated as in the original publication (see Materials and Methods). Shown is the resultant cluster UMAP and annotations, with BFB4 and BFB5 corresponding to brain fibroblast1 and brain fibroblast5. The clusters labelled leptomeninges correspond to BFB1-3 in Pietilä et al. (2023; Fig. 1D in that paper). (B, C) The transcriptomes shown in (A) were merged with the leptomeningeal and dural cell transcriptomes from Fig. 1E, run through our pipeline, and two rounds of Harmony batch correction performed. Shown are the resultant UMAPs, annotated for cell types (B) or for dataset of origin (C). Leptomeningeal clusters 0 and 4 are annotated based upon analyses included in this manuscript. The other clusters are comprised of transcriptomes from Pietilä et al. (2023), and are thus annotated as in that paper. (D) Violin plot showing the number of transcripts per cell (nCount) in each of the datasets included in the merged dataset in (B, C). Each dot represents an individual transcriptome. (E) UMAP as in (B) overlaid for expression of two mRNAs, Aldh1a2, and Crabp2 known to be expressed in the developing leptomeninges. Expression levels are color-coded as per the adjacent keys. (F) UMAP cluster visualization of the leptomeningeal transcriptomes from the Xenium mesenchymal probeset dataset (Fig. 4C), shown at higher resolution and annotated for potential pial versus arachnoid cells. (G) Violin plots showing relative expression levels of two pial mRNAs, Lamc3 and Bmp2 and two arachnoid mRNAs, Bnc2 and Dpp4 in the pial versus arachnoid cells as annotated in (F). Red and green denote arachnoid and pial cells, respectively. Each dot corresponds to expression in an individual cell. (H) Spatial plots of the cortical interface and midline showing distribution of the pia [file eneuro-12-ENEURO.0046-25.2025-s013.tif]

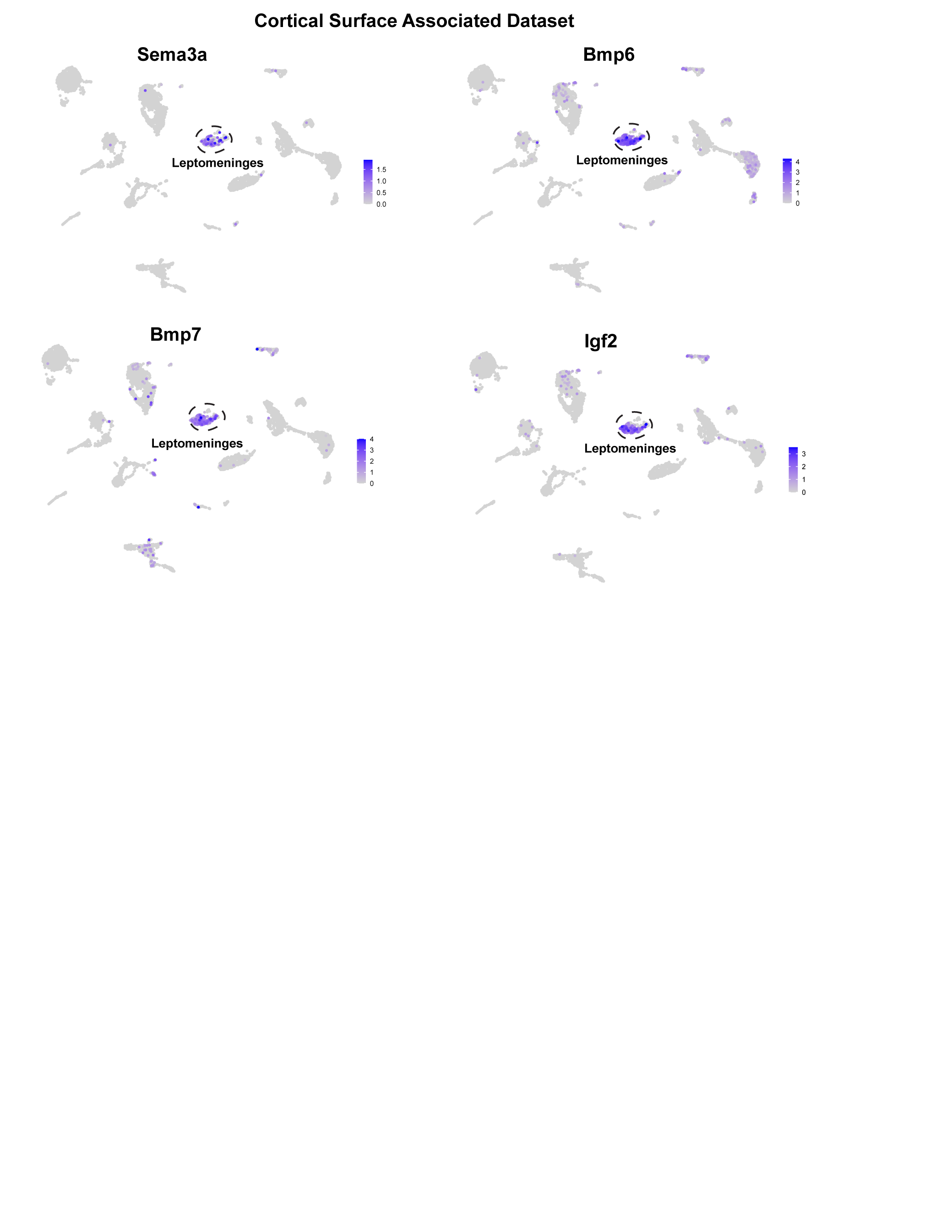

Supplement: Figure 8-1 — Specific expression of leptomeningeal ligands, as analyzed by scRNA-seq. UMAPs showing the cortical surface-associated dataset from Fig. 1A, overlaid for expression of four ligands that are highly-enriched in leptomeningeal cells, Sema3a, Bmp6, Bmp7 and Igf2. Expression levels are color-coded as per the adjacent keys. Download Figure 8-1, TIF file. [file eneuro-12-ENEURO.0046-25.2025-s015.tif]
